# Bio-Ecological Indicators for *Gentiana pneumonanthe* L. Climatic Suitability in the Iberian Peninsula

**DOI:** 10.3390/plants14182857

**Published:** 2025-09-12

**Authors:** Teresa R. Freitas, Sílvia Martins, Joaquim Jesus, João Campos, António Fernandes, Christoph Menz, Ernestino Maravalhas, Helder Fraga, João A. Santos

**Affiliations:** 1 Centre for the Research and Technology of Agroenvironmental and Biological Sciences, CITAB, Inov4Agro, University of Trás-os-Montes e Alto Douro, UTAD, Quinta de Prados, 5000-801 Vila Real, Portugal; silviamartins@utad.pt (S.M.); jjesus@utad.pt (J.J.); acpf91@utad.pt (A.F.); hfraga@utad.pt (H.F.); jsantos@utad.pt (J.A.S.); 2 Laboratory of Fluvial and Terrestrial Ecology, LEFT, University of Trás-os-Montes e Alto Douro, UTAD, Quinta de Prados, 5000-801 Vila Real, Portugal; 3 Centro de Investigação em Biodiversidade e Recursos Genéticos, CIBIO, InBIO Laboratório Associado, Universidade do Porto, Campus de Vairão, 4485-661 Vairão, Portugal; jc_campos@cibio.up.pt; 4 BIOPOLIS Program in Genomics, Biodiversity and Land Planning, CIBIO, Campus de Vairão, 4485-661 Vairão, Portugal; 5 Potsdam Institute for Climate Impact Research e. V., PIK, Telegrafenberg A 31, 14473 Potsdam, Germany; christoph.menz@pik-potsdam.de; 6 Centro de Conservação das Borboletas de Portugal, TAGIS, Rua das Portas de Évora, 3, 7480-152 Avis, Portugal; emsmaravalhas@gmail.com

**Keywords:** future climate conditions, marsh gentian, species conservation, worldwide bioclimatic classification system

## Abstract

*Gentiana pneumonanthe* L., a wetland specialist and exclusive host of the Alcon Blue (*Phengaris alcon*), is highly vulnerable to climate change. This study assessed the future climate suitability of the Iberian Peninsula (IP) for *G. pneumonanthe*. From 14 bioclimatic variables (ISIMIP3b, processed by CHELSA method at 1 km^2^) and two topographic variables, four bio-ecological indicators were selected using Pearson correlation and Variance Inflation Factors: Thermicity Index, Ombrothermic Index, Accumulated summer precipitation from June to August, and Maximum of the daily maximum temperature of August. A species distribution model platform (Biomod2) was applied for historical (1995–2014) and future periods (2041–2060, 2081–2100) under two anthropogenic radiative forcing scenarios (SSP3-7.0, SSP5-8.5). The ensemble model created shows a strong predictive performance (BOYCE: 0.98). Historically, 13.4% of the IP was climatically suitable, mainly in mountain areas. Under SSP3-7.0, suitable areas are projected to decline by 74.2% (2041–2060) and 99.3% (2081–2100); under SSP5-8.5, by 75.5% and 99.9%, respectively. While small gains may occur in the Pyrenees, most conservation protected areas (Natura 2000, RAMSAR) may lose suitability for species persistence. Such losses could disrupt ecological ecosystems and directly threaten the survival of *P. alcon*. These findings highlight the urgent need for climate-informed land-use planning and effective habitat conservation.

## 1. Introduction

*Gentiana pneumonanthe* L., commonly known as marsh gentian, is an herbaceous perennial hemicryptophyte widely distributed across Europe, Central Siberia and the Caucasus regions [1]. It predominantly inhabits montane and colder areas of the northern Mediterranean basin countries [2,3]. Within the Iberian Peninsula (IP), the species is found mainly in the northern regions and higher elevations (i.e., “Serra da Estrela” and “Sierra Nevada”) [2]. Marsh gentian grows in habitats with relatively high water levels, including lowland, heathlands, peaty marshes, peatland, grasslands, swamps and open wet forests [3,4]. Generally, these areas are defined by high annual precipitation, approximately a total precipitation of 1400 mm, and a mean temperature of 10 °C [5]. The species often grows along paths created by livestock or humans and in areas that are regularly mown [4]. This species is considered a ‘flagship species’ of wetland areas, used to raise awareness and promote conservation efforts [6]. It grows in protected areas designated under Natura 2000 and RAMSAR [7]. Natura 2000 intends to protect Europe’s biodiversity by conserving habitats and species of community interest [8]. Furthermore, the species exhibits a high level of interaction with the Alcon Blue (*Phengaris alcon*), as the unique host plant of this endangered butterfly species [9]. Female butterflies lay their eggs on young flower buds, and the caterpillars feed inside the fruit [10]. Once mature, the caterpillars drop to the ground and are collected by *Myrmica* ants, which then care for them to continue the development cycle. However, this reproductive cycle may be jeopardised by climate change, which can affect the phenology, development, and persistence of the marsh gentian in wetlands. Such changes may limit oviposition sites and larval food sources for the butterfly [11].

Despite the species’ high ecological representativeness in terms of habitat specificity and its essential function in the life cycle of the Alcon blue, the species is facing increasing challenges to its viability. The International Union for Conservation of Nature (IUCN) list marsh gentian as “Least Concern” in the “IUCN Red List of Threatened Species”, while also noting a decline in the number of its population over time [2,3]. *G. pneumonanthe* habitats are increasingly threatened by human activities, including the widespread draining of wetlands, soil eutrophication, declining groundwater levels, and changes in traditional small-scale agricultural land, such as the reduction in grazing. These forms of habitat deterioration may result in reduced offspring fitness and species conservation [4]. These anthropogenic pressures, combined with climate change, may act synergistically to intensify negative impacts on the species, affecting viability, reproduction, and vegetative growth [12,13,14,15]. In the Mediterranean region, particularly in IP, annual precipitation has decreased while temperature and aridity have increased [16,17]. These trends raise concerns about the stability of the ecosystem when this species grows, compromising its survival [5]. Additionally, drought and water shortages are becoming more frequent and intense [5,16]. Although *G. pneumonanthe* has been studied in Central and Northern Europe (e.g., [10]), knowledge about its climatic response under future scenarios remains limited, particularly in our study area, where climatic conditions impose specific ecological constraints. Understanding climate trends is essential for assessing potential impacts on plant species and developing effective conservation measures. Shared Socioeconomic Pathways (SSPs) [18] provide projections of worldwide socioeconomic trends through development until 2100, which can be utilised to simulate (and are usually applied to create) scenarios for future greenhouse gas emission concentrations under several different climate policies [19,20]. These pathways help to provide a comprehensive understanding of climate change, from the past to the future.

Species distribution models (SDMs) are widely used tools with practical applications to predict biodiversity patterns. They help clarify how the environmental factors (i.e., geography, soil and climate) influence the distribution of the species while also identifying potential suitable habitats under current and future climate change scenarios [21,22]. SDMs are commonly used to interpret the probability of species occurrence, assess habitat suitability, and project how these conditions may shift with changing climate and landscape scenarios [19,23]. Correlative and statistical models, in particular, use different algorithms (e.g., Generalized linear models (GLM), Random forests (RF), Classification tree analysis (CTA)) to determine the influence of the environment [24]. Moreover, the application of these models can support the development of guidelines for mitigation and adaptation strategies for species conservation under climate change [21,23]. However, it is crucial to acknowledge their high sensitivity to input data (species occurrence points) and modelling parameters, particularly predictor selection and sampling bias, which can affect model reliability [25,26]. Careful consideration of these factors is essential to ensure that SDMs reliably support conservation planning and decision-making, particularly for species of conservation concern [24].

The present study aims to (1) determine the bio-ecological indicators that influence the viability and growth of marsh gentian; (2) identify the suitable regions for the continuance and viability of the species; (3) assess the future suitability, and consequent future changes, regarding protected areas (the Nature 2000 and the RAMSAR). To achieve these goals, we identify the bio-ecological parameters most relevant to the species’ development to construct an accurate and robust SDM ensemble. Using the Biomod2 platform, we develop the distribution ensemble model for marsh gentian and provide the principal changes in future conditions for SSP3-7.0 and SSP5-8.5 for two periods, 2041–2060 and 2081–2100. Based on these projections, the study discusses adaptation strategies to support species conservation and explores the potential implications for protected areas.

## 2. Results

### 2.1. Bio-Ecological Indicators and Gentiana pneumonanthe Distribution

**Figure 1:**
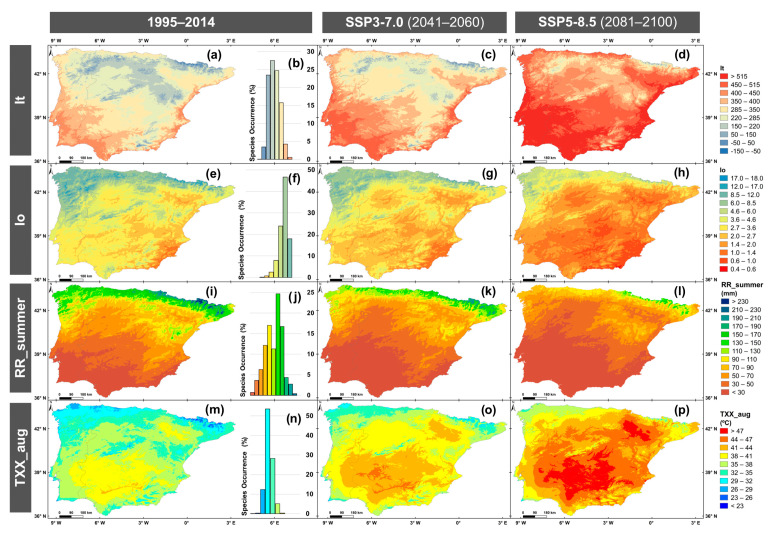
Ensemble means bio-ecological indicators for the historical period (1995–2014) and future scenarios: SSP3-7.0 (2041–2060) and SSP5-8.5 (2081–2100). The maps show: Thermicity index (It; (**a**,**c**,**d**)), Ombrothermic index (Io; (**e**,**g**,**h**)), Accumulated summer precipitation (June–August) in mm (RR_summer; (**i**,**k**,**l**)), and Maximum of the daily maximum temperature of the hottest month (August) in °C (TXX_aug; (**m**,**o**,**p**)). Marsh gentian occurrence percentages (%) are illustrated concerning the bio-ecological indicator: It (**b**), Io (**f**), RR_summer (**j**), and TXX_aug (**n**). The threshold values for the thermotypes and ombrotypes horizons are defined by Rivas-Martínez [27].

The ensemble means of bio-ecological indicators, Thermicity Index (It), Ombrothermic Index (Io), Accumulated summer precipitation from June to August (RR_summer), and Maximum of the daily maximum temperature of the hottest month (August) (TXX_aug), are represented in Figure 1 for historical and future periods: 2041–2060 (SSP3-7.0) and 2081–2100 (SSP5-8.5). The results indicate that the patterns of these indicators are highly similar across scenarios and periods. To avoid redundancy, additional projections for SSP5-8.5 (2041–2060) and SSP3-7.0 (2081–2100) are provided in the supplementary material (Figure S1), which show spatial and temporal trends.

During the historical period, the upper supramediterranean thermotype (−150–150 It), as defined by the Rivas-Martínez thermotype horizons (Table S1), located in the Pyrenees, accounted for 4.5% of the territory, while the lower supramediterranean (150–220 It) is found in the Iberian and Central Systems and the Cantabrian Mountains (covering 17.06%) (Figure 1a). At lower elevation and the central IP, the upper (220–285 It) and lower (285–350 It) mesomediterranean is observed, covering 28.7% and 26.8%. of the IP, respectively. The thermomediterranean areas were mostly observed in coastal and southern IP, with the upper (350–400 It) making up 15.9% of the area, and the lower (400–450 It) 7.0%. The upper inframediterranean (450–515 It), present in the Marbella region, covered 0.06% of the area. Marsh gentian occurrences (Figure 1b; Table S2) were mainly associated with the supramediterranean (54.6%), mesomediterranean (40.5%), and thermomediterranean (4.9%) thermotypes. By 2041–2060 (SSP3-7.0; Figure 1c), the supramediterranean is expected to persist only in high elevation areas (5.3% of the total), while the inframediterranean is expected to expand by 11.8%, especially in the southwestern and eastern IP. By the 2081–2100 period (SSP5-8.5; Figure 1d), compared to the historical period, the supramediterranean and mesomediterranean are projected to decline by 16.7% and 48.3%, respectively. In contrast, the thermomediterranean and inframediterranean are expected to increase by 13.7% and 55.9%, respectively. These results indicate that the species develops primarily in cooler areas. As It increases in future, marsh gentian is likely to be exposed to higher temperatures, which could negatively affect its growth and phenological cycle.

Regarding Io, during the historical period (Figure 1e), lower (1.0–1.4 Io) and upper (1.4–2.0 Io) semiarid ombrotype (Table S3) were observed in the southeastern IP, covering 0.9% and 2.8%, respectively. Except in the northwestern IP, lower (2.0–2.7 Io) and upper (2.7–3.6 Io) dry areas were widespread, covering 14.0% and 31.8%, respectively. At higher elevations, subhumid areas were more prevalent, with the lower (3.6–4.6 Io) representing 19.6% of the area and the upper (4.6–6.0 Io) 10.9%. In the northern and northwestern IP, humid conditions were found, with the lower (6.0–8.5 Io) covering 10.8% and the upper (8.5–12.0 Io) 8.0%. The hyperhumid (12.0–24.0 Io), though limited to 1.3% of the area, was observed in high mountain regions (i.e., the Pyrenees, the Cantabrian Mountains, and the Galician Massif). Species occurrences (Figure 1f; Table S2) were found predominantly under dry (3.3%), subhumid (31.8%), and hyperhumid (64.7%) ombrotypes. In the medium-term (SSP3-7.0; Figure 1g), projections indicate an increase in semiarid and dry areas by 10.2% and 10.6%, respectively, especially in the southern, central, and eastern IP. In contrast, subhumid, humid, and hyperhumid areas are expected to decline by 13.5%, 6.1%, and 1.6%, respectively, particularly in the northwestern IP. By the long-term (SSP5-8.5; Figure 1h), hyperhumid areas are projected to disappear, and humid areas will become residual (0.84% of the territory). Semiarid ombrotype is expected to increase by approximately 57.05%, covering most of the southern, central, and eastern IP. In addition, arid ombrotype is projected to emerge, occupying around 7.3% of the southeastern IP.

As for RR_summer, during 1995–2014 (Figure 1i), lower precipitation was observed in the southern compared with the northern regions. The lowest precipitation (<30 mm) covered 18.7% of the area, whereas the highest precipitation (>190 mm) was recorded in the Pyrenees, accounting for 1.2% of the territory. Across the northern IP, including northwest Portugal and the Iberian System, RR_summer range 90–190 mm. Marsh gentian occurrence is below 10.6% in areas with low (<70 mm) and high precipitation (>170 mm) (Figure 1j; Table S2). The highest occurrence was found in intermediate precipitation levels: 12.1% in areas receiving 70–90 mm, 17.0% in 90–110 mm, 11.3% in 110–130 mm, 24.7% in 130–150 mm, and 17.0% in 150–170 mm. Under the 2041–2060 period (Figure 1k), precipitation above 210 mm becomes marginally covered (1.8% cover area). The area receiving only <30 mm is projected to increase by 9.1%. In northern IP, northwest Portugal, and the Iberian System, 90–170 mm (17.1% of the total area) will be recorded. By 2081–2100 (Figure 1l), low summer precipitation (<70 mm) is expected to affect 12.0% more of the land compared to the historical period. Only the Pyrenees will still receive 90–150 mm (5.8% of the area), while the northwest of Portugal and the Iberian System will record 50–110 mm (31.1%).

In the historical period, TXX_aug (Figure 1m) ranges from < 23 °C, in the Pyrenees, covering less than 0.5% of the area. In the Pyrenees, Guadarrama Mountains and along the coast (13.5% of the area), temperatures range from 23–26 °C, 26–29 °C, and 29–32 °C. In contrast, temperatures between 38–44 °C were recorded across central and southern regions of IP, particularly in the “Sierra Morena” and Ebro River depression (22.4% of the total area). TXX_aug between 41–44 °C occur in the Guadalquivir River depression (0.6% of the area). The species (Figure 1n; Table S2) was predominantly found in regions with TXX_aug between 26–29 °C (12.4% of the occurrence area), 29–32 °C (53.5%), and 32–35 °C (28.3%). In the medium-term (Figure 1o), TXX_aug 23–32 °C is expected to persist in the Pyrenees, as well as in the northwest, southwest, and Guadarrama Mountains, covering 1.5% of the total area. Conversely, in the Ebro River depression and south of the Central System, particularly in the areas surrounding the Guadalquivir and Guadiana rivers, temperatures are expected to reach 47 °C. In the long-term (Figure 1p), new temperature extremes above 47 °C are projected, potentially affecting 14.3% of the territory. The lowest temperatures (26–35 °C) will continue to be recorded along the coast, especially in the western and northern IP and in the Pyrenees (1.0% of the area).

In terms of the It, values in 2041–2060 under SSP5-8.5 (Figure S1a) closely resemble those under SSP3-7.0. For 2081–2100, SSP3-7.0 (Figure S1b) is projected to have a greater extent of areas with lower It values. Concerning Io, 2041–2060, SSP5-8.5 (Figure S1c) shows upper arid ombrotype, which covers 0.5% of the IP. During 2081–2100, SSP3-7.0 (Figure S1d) presents less area (4.1%) of arid ombrotype, compared with SSP5-8.5. Regarding RR_summer, in the 2041–2060 period, SSP5-8.5 (Figure S1e) is projected to have more extensive areas receiving only 10–30 mm, and by 2081–2100, SSP3-7.0 (Figure S1f) will show a lower precipitation overall compared to SSP5-8.5. In terms of TXX_aug, coastal temperatures remain lower under SSP5-8.5 in 2041–2060 (Figure S1g). However, by 2081–2100, SSP3-7.0 (Figure S1h) is projected to have slightly lower temperatures compared to SSP5-8.5. The differences between SSP3-7.0/SSP5-8.5 in 2041–2060 and 2081–2100 are in the same order of magnitude as the natural variability.

### 2.2. Accuracy Evaluation of SDMs

Under current climate conditions, model evaluation was conducted for six algorithms: CTA, GA, GBM, GLM, MARS and RF (Table S4). The mean performance of these algorithms was compared according to TSS and BOYCE metrics during calibration (Figure S2a) and validation (Figure S2b). Overall, the BOYCE showed a better value compared to TSS, indicating better performance in capturing the interaction between species distribution and bio-ecological conditions. Furthermore, BOYCE is a spatial metric that assesses model predictions based on spatial accuracy. Consequently, BOYCE above 0.8 was used to build the ensemble model (EM).

The resulting EM was evaluated by the metrics TSS, AUCroc, BOYCE, Bias and CSI (Table 1). TSS, AUCroc, and BOYCE revealed that the model has a high capacity to predict marsh gentian occurrences (sensitivity), while BIAS and CSI reveal a lower predictive capacity. Regarding specificity, how well the model predicts the pseudo-absence of occurrences, all the metrics showed values above 0.8. In terms of calibration, AUCroc, BOYCE and Bias showed high values (>0.9), which means that the models had algorithms a higher performance, while TSS indicated moderate calibration (0.7) and CSI lower performance (0.4).

The response curves of individual models and EM are similar, with Io representing the most important variable (Figure S3a,e), followed by TXX_aug (Figure S3d,h) and It (Figure S3b,f), and with RR_summer (Figure S3c,g) contributing the least. The Io combines precipitation and temperature in its calculation, providing a more integrative measure of water availability. This allows a better description of the species’ distribution, as it captures the key ecological processes underlying habitat suitability.

The presence probability (>0.6) is higher when the It (Figure S4a) is negative (It <−100). The probability then decreased, stabilising between 0.1 and 0.2 from 0 to 390 It, before increasing again beyond 390 It. For Io (Figure S4b), the occurrence probability increased with higher Io values, peaking at 0.8 corresponding to 12 Io. Regarding RR_summer (Figure S4c) and TXX_aug (Figure S4d), the occurrence probability is less than 0.3, with the highest values recorded below 50 mm of rainfall and between 27 °C and 33 °C, respectively.

### 2.3. Climatic Suitability for Gentiana pneumonanthe

**Figure 2:**
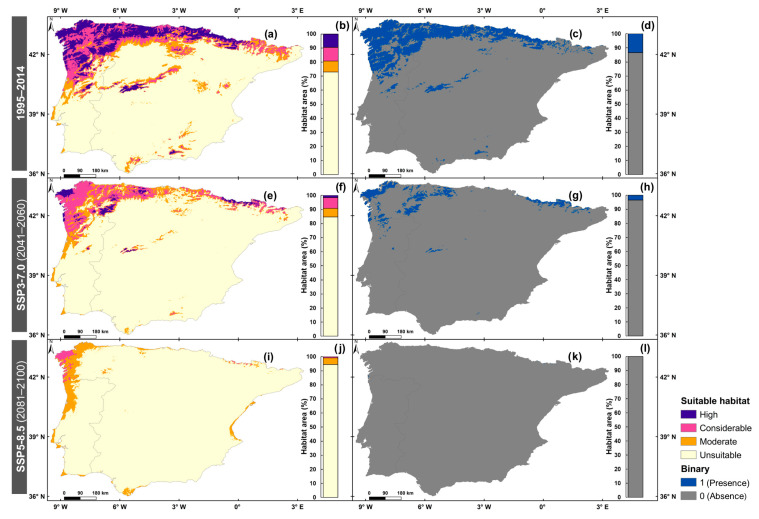
Climatic suitability classes (high, considerable, moderate and unsuitable) and binary distribution (1 = presence and 0 = absence) are represented for the historical period 1995–2014 (**a**,**c**) and future scenarios: SSP3-7.0 for 2041–2060 (**e**,**g**) and SSP5-8.5 for 2081–2100 (**i**,**k**). The corresponding climatically suitable area percentages, based on suitability classes and binary classification, are presented for 1995–2014 (**b**,**d**), 2041–2060 (**f**,**h**) and 2081–2100 (**j**,**l**).

According to the predicted climatic suitability for marsh gentian in the historical period (Figure 2a), 72.8% of the IP (mostly in the south, east, and central regions) is classified as unsuitable (Figure 2b). Moderate suitability, covering 7.8% of the total area, particularly in western Portugal and around the Malaga region (southern Spain). As the elevation increases, areas with considerable suitability (9.9%) appear, particularly in the Pyrenees, northern IP and northwestern Portugal. High suitability is observed mainly in the northern IP and northwestern Portugal (9.4% of the area). This class is also observed in high elevation areas, namely “Serra da Estrela”, the Central System and “Sierra Nevada”. The binary maps (Figure 2c) show that 13.4% of the area (Figure 2d) is suitable for marsh gentian development. When intersecting the continuous suitability and binary classification, only the areas categorised as high and considerable correspond to the binary suitability.

For the 2041–2060 period (SSP3-7.0) (Figure 2e), “Sierra Nevada” no longer has high suitability and “Serra da Estrela” and the Central System have reduced area corresponding to this class. Furthermore, in northern IP and northwestern Portugal, there has been an increase in considerable suitability and a decrease in high suitability areas. Although the Pyrenees show a reduction in moderate and considerable suitability, there is an increase in high suitability. Compared with the historical simulations, unsuitable areas increase by 11.9% (Figure 2f), while moderate, considerable and high suitability areas decrease by 1.8%, 2.3% and 7.9%, respectively. In the binary analysis (Figure 2g), only 3.5% of the IP (Figure 2h) is classified as suitable, which corresponds only to areas of high or considerable suitability.

For the 2081–2100 period (SSP5-8.5) (Figure 2i), high suitability areas will disappear. A considerable suitability area is observed in northwestern IP and the Pyrenees (0.8% of the area) (Figure 2j). A moderate suitability area is observed along the coast (4.7%), with a predominance in the western IP. The remaining 94.4% of the IP is classified as unsuitable. The binary data (Figure 2k) indicates that only 0.1% of the area (Figure 2l) is suitable for marsh gentian, and this corresponds to the considerable suitability class. This intensive reduction indicates a severe contraction of available habitat, posing a substantial extinction risk for *G. pneumonanthe* in the study region.

Under the SSP5-8.5 (2041–2060; Figure S5a), unsuitability areas increase by 16.0% compared to the historical period (Figure S5b). According to the binary suitability maps (Figure S5c), only 5.7% of the IP (Figure S5d) is suitable for marsh gentian, corresponding to regions classified as high, considerable, or moderate suitability. For the SSP3-7.0 (2081–2100; Figure S5e), habitat suitability ranges between considerable and moderate, covering 4.9% of the area (Figure S5f). However, only 0.1% of the area is classified as suitable according to the binary map (Figure S5g,h).

### 2.4. Dynamic Changes in Areas of Climatic Suitability

**Figure 3:**
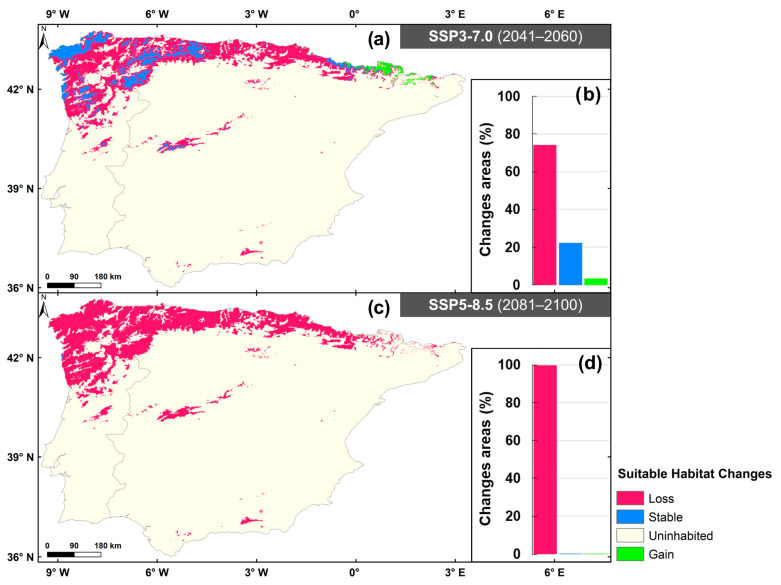
Dynamic changes in suitable areas between historical and future periods are illustrated. The changes are represented according to three categories: loss, stable and gain. These spatial distributions are represented in the maps, while their area percentage are presented in the graphs. Panels (**a**,**b**) correspond to SSP3-7.0 for 2041–2060, and panels (**c**,**d**) correspond to SSP5-8.5 for 2081–2100.

The dynamics of climatic suitability based on the changes between historical and future periods are illustrated in Figure 3. For the 2041–2060 period (SSP3-7.0), there is a significant loss of 74.2% of previously suitable areas (Figure 3a,b). However, 22.4% of the suitable areas remain stable, particularly in the Pyrenees, northwest of IP and high elevation areas, such as “Serra da Estrela”, Central System and “Sierra Nevada”. Additionally, a 3.5% gain in suitable areas is observed in the Pyrenees, where future climatic conditions are expected to improve the development of marsh gentian. For the 2081–2100 period (SSP5-8.5) (Figure 3c,d), 99.91% of the suitable habitat present during the historical period is lost. Only in the northwest of IP, between Viana do Castelo and Vigo cities, areas show that maintain a suitable habitat (0.04%). A minor gain in the suitable area of 0.05% is detected between the “Ordesa y Monte Perdido National Park” and the Aneto mountain.

Under the SSP5-8.5 (2041–2060; Figure S6a,b), 20.1% of the area remains suitable, with an additional 2.4% gained. In contrast, under the SSP3-7.0 (2081–2100; Figure S6c,d), only 0.4% of the suitable area is maintained, and 0.4% is newly gained. Although some areas are projected to become newly suitable for marsh gentian, successful colonisation will depend on additional factors, i.e., seed dispersal and soil type, which may limit the species’ establishment.

### 2.5. Impact of Conservation Areas on Species Viability

*G. pneumonanthe* is one of the target species for conservation within the Natura 2000 and Ramsar conservation areas. Therefore, understanding how climate change may affect the suitability of these protected areas for the species is essential.

**Figure 4:**
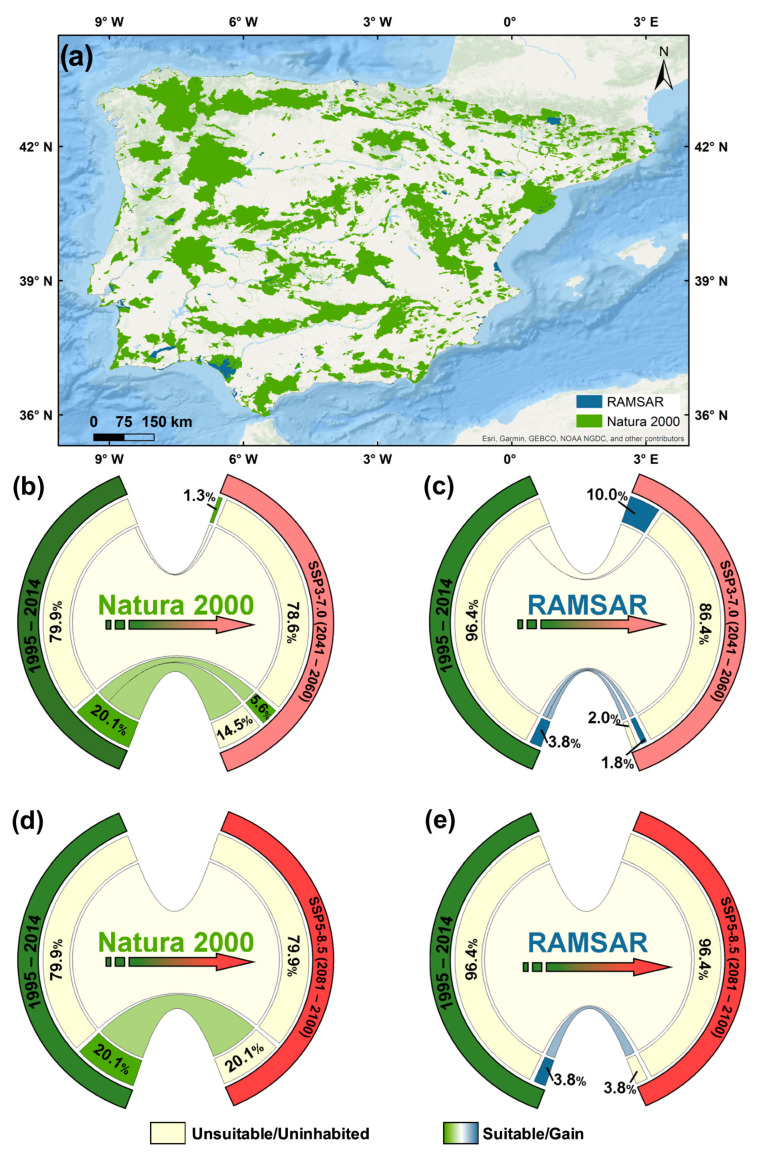
Natura 2000 and RAMSAR distribution in the Iberian Peninsula (**a**). Quantification of changes in suitability within Natura 2000 (**b**,**d**) and RAMSAR (**c**,**e**) areas, from the historical (1995–2014) period to future scenarios: SSP3-7.0 (2041–2060) and SSP5-8.5 (2081–2100).

During historical simulations (Figure 4a), only 20.1% of the Natura 2000 area had climate conditions suitable for the species. Under the SSP3-7.0 scenario for 2041–2060 (Figure 4b), projections indicate that 14.5% of this optimal area could be lost and 5.6% conserved. In terms of unsuitable areas, 1.3% of these areas will be converted to suitable ones. In this future period, 6.9% of Natura 2000 will be suitable for the species. Regarding RAMSAR (Figure 4c), 3.8% of the area was suitable for the species in the historical period. In the medium-term, 10.0% of the previously unsuitable areas become favourable for marsh gentian, resulting in a total of 11.8% of Ramsar areas with suitable conditions. However, for the period 2081–2100, projections under SSP5-8.5 indicate that none of the areas within Natura 2000 (Figure 4d) and RAMSAR (Figure 4e) will offer climate conditions suitable for the development of marsh gentian. In contrast, projections under SSP3-7.0 for the same period show that small gains are still possible, with 0.1% of Natura 2000 (Figure S4d) and 0.9% of Ramsar areas (Figure S4e) expected to transition from unsuitable to suitable conditions. The severe decline in suitable areas underscores the urgent need to integrate climate adaptation strategies into the management of protected areas to preserve *G. pneumonanthe* habitats.

## 3. Discussion

The IP was historically characterised by climates ranging from supramediterranean to inframediterranean, according to the Thermicity index [27], which agrees with previous studies [28,29,30]. *G. pneumonanthe* predominantly occurred in supramediterranean (54.6%), mesomediterranean (40.5%), and thermomediterranean (4.9%) climates. Under the SSP5-8.5 (2081–2100), projected temperature increases suggest that 45.2% and 20.1% of these areas may shift to thermomediterranean and inframediterranean climates, respectively. Regarding ombrotypes [27], IP has historically ranged from dry to hyperhumid climates. Marsh gentian has primarily been found in hyperhumid (64.7%), subhumid (31.8%), and, to a lesser extent, dry (3.3%) areas. However, under SSP5-8.5 (2081–2100), its historical habitats are expected to shift towards arid conditions [29] with a total area of 3.3% semiarid, 18.2% dry, 67.5% subhumid, and only 10.9% hyperhumid. In terms of RR_summer and Txx_Aug, the species typically occurred in areas with 30–210 mm and 26–35 °C, respectively. Under SSP5-8.5 (2081–2100), RR_summer is expected to decrease (30–150 mm) and Txx_Aug increases (35–44 °C). Studies on related species (*G. lutea*) indicate a sharp decline in germination above 20 °C, with adverse effects at 25–30 °C [31]. These findings suggest that rising temperature extremes may exceed critical thresholds for gentian development. Since 1998, habitat loss (i.e., heathlands) and negligence have been recorded in several regions, such as the Netherlands and the United Kingdom [4,12,32]. The present study integrates the latest anthropogenic radiative forcing scenarios (SSPs) [18,33] and the data at a high spatial resolution (1 km), allowing for more detailed and accurate analysis [20,34].

Several studies have successfully applied SDMs, particularly through the Biomod2 platform [19,21,35,36,37]. The BOYCE was selected (calibration = 0.98) as the main evaluation/validation metric and the objective function for calibration, as it measures model performance using spatial parameters [22,36]. The resulting model accurately identified suitable areas distribution for *G. pneumonanthe* in IP, identifying the Io (70% of importance) as the most influential indicator, followed by the TXX_aug (16%), It (10%), and RR_summer (6%). Historically, higher elevation areas (i.e., “Serra da Estrela”, Central Systems and “Sierra Nevada”) and the northern IP showed greater suitability for the species, covering 13.4% of the IP. However, suitable areas are projected to change in future climates. Under SSP3-7.0, it is expected to decrease by 74.17% in 2041–2060 and by 99.28% in 2081–2100. By SSP5-8.5, the reduction is projected at 75.53% for 2041–2060 and 99.91% for 2081–2100. However, new suitable areas may emerge, particularly in the Pyrenees, potentially providing future suitable areas for gentian marsh growth. In SSP3-7.0, new suitable areas could reach 3.45% in 2041–2060 and 0.35% in 2081–2100. Under SSP5-8.5, projections are 2.40% and 0.05% for the same periods. Nonetheless, it is important to note that these areas remain very limited in extent, and successful colonisation will depend on the presence of appropriate ecological conditions and species interactions in these new locations [38]. These results underscore the significant impact of climate on the distribution and development of plant species. However, it is important to consider some SDM’s limitations [39,40]. These models are dependent on observed occurrence data, which may not reflect the full potential distribution of a species or contribute to model uncertainty if sampling bias occurs [25,26]. It is recommended to use at least five predictor variables to improve model reliability. Although the use of at least five predictor variables is generally recommended, this study employed four bioclimatic predictors, including the composite indices Io and It. These indices aggregate multiple underlying climate variables [28,29,30]. Their integrative nature enhances explanatory power, supporting model reliability. Furthermore, future research should incorporate additional variables such as soil properties, land use, and human disturbance to complement and refine the results obtained.

Suitable conditions for the marsh gentian’s growth have been declining over time, leading to the species being confined to increasingly unfavourable environments [4,12,41]. With projected temperature and aridity increases and precipitation decline, the species’ development, propagation, germination, and flowering viability are expected to change [12]. Additionally, the high variability of rainy-day frequencies can also impact pollen transfer, as functional flowers remain closed during such periods, and reduce seed production [42]. Additionally, periods of drought or excessive rainfall may increase mortality rates [12]. Small and fragmented populations of marsh gentian are particularly vulnerable to climate change, especially due to reduced pollination rates, lower seedling recruitment, and a decline in genetic diversity, all of which contribute to the risk of extinction [6,9]. The loss of marsh gentian would have wider ecological consequences, resulting in biodiversity loss, particularly affecting its complex interaction with *Phengaris alcon* and *Myrmica* ants [43]. As the marsh gentian is the unique host plant for the eggs and caterpillars of the rare Alcon Blue (*Phengaris alcon*), its extinction would endanger the butterfly’s survival [9,11]. Beyond this specific interaction, marsh gentian also contributes to broader wetland ecosystem functions, including maintaining habitat structure and supporting biodiversity [1]. Marsh gentian has been used in herbal medicine to treat respiratory conditions, digestive and liver disorders [44]. Hence, its extinction in the IP would not only represent a significant ecological loss but also a potential setback to public health and the economy. Furthermore, the management plans for protected areas (such as Natura 2000 and RAMSAR) currently lack procedures that address nature conservation in the context of projected future climate change [45]. Nevertheless, the European Parliament and the Council have established legally binding targets and obligations aimed at restoring degraded habitats and combating biodiversity loss by 2050 [46,47]. This underscores the urgency of linking ecosystem services, such as those provided by marsh gentian, to policy and conservation action.

Although multiple parameters can influence the viability of the species, as biotic interactions (i.e., reproductive success, genetic variation, population dynamics, nutrient availability) and abiotic factors (i.e., mowing, grazing, and sod-cutting) [1,41,48], which are not addressed in the present study, it is important to recognise that maintaining a species under hostile climatic conditions is a significant challenge. One possibility for the species’ persistence under such conditions is through natural adaptation or genetic improvement [4]. Artificial inter-population crossing may support the conservation of the species within its current habitats and also facilitate its propagation into new areas, where future climatic conditions may become more favourable. Seedling recruitment, seedling growth potential, and seed production could be utilised to support the relocation and establishment of the species in new habitats [48]. Active management strategies, grazing and controlled burning, are also recommended, as they help maintain the open, wet conditions essential for marsh gentian flowering and seedling establishment [6,32]. Studies have shown that practices such as late mowing or moderate grazing help maintain open habitats and favour the recruitment of wetland species, whereas the absence of management often leads to habitat closure and population decline [41,49]. However, although suitable areas are projected to emerge in the Pyrenees, suggesting that relocating the species to higher altitudes could serve as a potential adaptation measure [20], relocation would need to align with existing European Union (EU) conservation policies, which prioritise in situ protection. However, relocation would need to align with existing EU conservation policies, which prioritise in situ protection [46] and are not explicitly regulated. It should therefore be regarded only as a complementary or last-resort measure. All these proposed strategies would therefore require careful evaluation and testing before practical implementation. Natural parks play a vital role in the conservation of the *G. pneumonanthe* and *P. alcon* through heathland management practices that include the measures mentioned earlier. Examples of this management can be found in the Groane Regional Park and the Briantea Heathland Park, in Italy [6], and at the Alvão Natural Park in Portugal [32]. The preservation of *G. pneumonanthe* directly contributes to the conservation of *P. alcon*, whose larvae depend exclusively on this plant for oviposition. Conservation measures must integrate both species, focusing on the protection and restoration of wet meadows, regulating grazing intensity, and adjusting mowing schedules to allow for seed dispersal and larval development [47,49,50].

This study opens new questions for forthcoming research. We recommend comparing mechanistic models with the SDM results obtained to evaluate consistency in predicted distribution. Future studies should also test whether the climatic variables identified in this study are applicable for modelling marsh gentian distribution in other regions of Europe. Given the strong interaction between the marsh gentian and the Alcon Blue, it is recommended that further research be carried out to assess the impact of climate change on the butterfly. We believe that combining existing research with new studies will enhance our understanding of the ecosystem that supports the viability and interaction of these species. This knowledge will contribute to more effective habitat management and species conservation, guided by Nature-based Solutions.

## 4. Materials and Methods

### 4.1. Description of Gentiana pneumonanthe L. Phenological Stages

**Figure 5:**
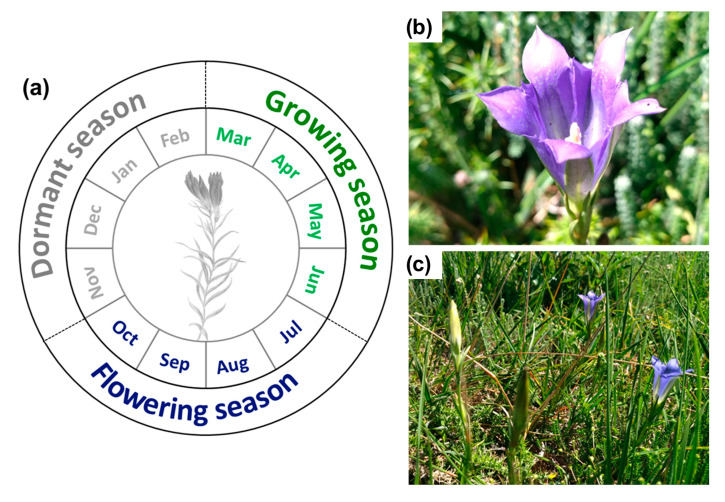
*Gentiana pneumonanthe* L. scheme of phenological stages (**a**), flowering stage (**b**) and habitat context (**c**) (photos taken by Silvia Martins).

Rose et al. [12] identified three phenological stages for marsh gentian (Figure 5a). In the dormancy phase, the plant overwinters below ground and retains the nutrients incorporated over the growing season [51]. After the dormancy phase, the growing season begins when the plants’ shoots start to emerge and develop. This is followed by the flowering stage (Figure 5b,c), characterised by the production of one or more flowering or non-flowering shoots. Mature reproductive plants typically develop multiple stems, each capable of bearing more than 30 deep violet-blue flowers, and reproduction occurs through seed production [52].

### 4.2. Study Area Description and Gentiana pneumonanthe L. Occurrence Records

**Figure 6:**
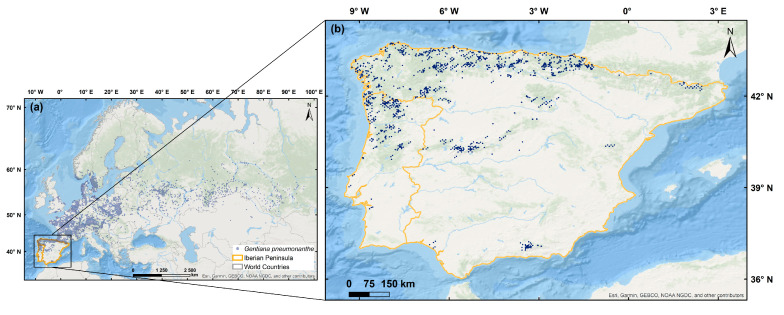
Occurrence records of *Gentiana pneumonanthe* L. across Eurasia (**a**) and within the Iberian Peninsula (IP) (**b**).

*G. pneumonanthe*, a Eurasian suboceanic species, is distributed across Europe, Central Siberia, and the Caucasus regions (Figure 6a) [1]. In Europe, it can be found from southern Scandinavia to northern Portugal and eastward into Russia [52]. The study area, which encompasses Portugal and Spain’s continental regions, exhibits a high diversity of climate conditions, soil parameters, and land use/land cover. To define the species distribution across IP, we utilised occurrence data from six databases (Table S5) [30,53,54,55,56]. Each dataset included geographic coordinates (longitude, latitude) for the marsh gentian. These databases were analysed simultaneously to identify and eliminate incorrect and ambiguous points, such as the occurrences recorded in the middle of the ocean. Records lacking precise geographic data or containing repeated entries were excluded [21,57]. The remaining occurrence points were recorded and mapped in decimal degree format to create a structured and comprehensive distribution database for *G. pneumonanthe* in the IP. In total, 2381 marsh gentian occurrence points are used in the present study (Figure 6b). To reduce model overfitting, these points were spatially filtered using the SpThin package in R software [19,58]. Duplicate records, representing the same or nearby individuals within a 1 km radius, were removed, ensuring that only one occurrence point per grid cell (1 × 1 km^2^) was retained [23,35]. This threshold was selected to align with the resolution of the climate data. This spatial thinning reduces sampling bias and spatial autocorrelation by preventing the model from being overly influenced by clusters of occurrence points, which can lead to unrealistic predictions based on localised environmental conditions [58,59]. After filtering, 1277 unique occurrences were reserved. The final dataset was stored as CSV files, which included longitude, latitude, and species name.

### 4.3. Climate Dataset

Daily climate simulations at 30 arc-seconds resolution from an ensemble of nine-member Global Circulation Models (GCMs) (Table S6) [60,61,62,63,64,65,66,67,68] were processed using the CHELSA method (version 2.1). This method [34,69] is applied to downscale global climate simulations, using a quasi-mechanistic framework, which enhances the spatial resolution of global climate models by incorporating physical processes such as orographic blocking, temperature stratification, and cloud and precipitation formation. The CHELSA method was applied to bias-adjust climate projections from the Inter-Sectoral Impact Model Intercomparison Project (ISIMIP3b; 0.5◦ grid resolution) [70,71,72,73], based on Coupled Model Intercomparison Project Phase 6 (CMIP6) simulations [74,75]. Two Shared Socio-Economic Pathways (SSPs) were applied. The SSP3-7.0 (regional rivalry), a moderately pessimistic scenario with a projected radiative forcing of 7.0 W/m^2^ by 2100 [76], and the SSP5-8.5 (fossil-fuelled development), a high-emissions trajectory with radiative forcing reaching 8.5 W/m^2^ in 2100 [77]. From the CHELSA method, daily data of precipitation and air temperature (maximum, minimum and mean) were obtained for each scenario (SSP) and GCM [78]. Each combination of scenario and model was used to calculate bio-ecological indicators, based on a 20-year ensemble mean [51]. These ensemble means were computed for the historical baseline (1995–2014) and future periods: medium-term (2041–2060) and long-term (2081–2100). Using a 20-year ensemble ensures ecological relevance by capturing long-term climate trends while minimising the influence of short-term variability. It is important to note that the historical baseline is derived from bias-adjusted climate simulations.

Given the high similarity in future periods 2041–2060 and 2081–2100 between scenarios, we chose to represent SSP3-7.0 for the period 2041–2060 and SSP5-8.5 for 2081–2100 in the main text, as these scenarios revealed the most distinct differences. In contrast, the results for the same periods under the alternate scenarios, SSP5-8.5 (2041–2060) and SSP3-7.0 (2081–2100), showed greater similarity and are therefore included in the supplementary material.

### 4.4. Bio-Ecological Indicators Related to Species Distribution

To assess the environmental factors influencing the distribution and viability of marsh gentian, we selected 16 bio-ecological parameters as candidate variables for SDM (Table S7), 14 bioclimatic indices and 2 topographic indicators. Bioclimatic indicators were based on air temperature and precipitation, which have several effects on the phenological development of the species [12,53]. Additionally, we incorporated widely used compound bioclimatic indices that combine multiple simple bioclimatic parameters: the Thermicity Index (It), Continentality Index (Ic), and Annual Ombrothermic Index (Io) [27,79,80]. To reflect the increasing frequency of extreme weather events projected for the region, we included the indicators: precipitation percent due to R95p days (R95PTOT), very warm days percent with respect to 90th percentile of reference period (TX90P), and the De Martonne Index (DMI) [17,81,82]. All the climatic parameters were calculated by the authors. Topographic variables (digital terrain model and slope) and soil type were also considered.

To determine the interaction among the 16 environmental variables, consistent with other studies [57], Pearson correlation analysis was first conducted. This analysis helps to reduce the risk of model overfitting [35]. Variables with a high correlation (|r| ≥ 0.7) were excluded (Figure S8). Although Slope and TX90P presented low correlation values, ecological and spatial distribution analyses indicated that these variables offered limited interpretability of *G. pneumonanthe* occurrence. Their weak alignment with the species’ known distribution patterns, compared with other predictors (e.g., Thermicity Index and precipitation [30]) with stronger ecological relevance, justified their exclusion from the final model. Afterwards, the Variance Inflation Factor (VIF) was applied to reduce multicollinear between selected variables [19,26]. Only the variables with VIF < 5 were included in the model. This threshold is widely applied in ecological and statistical modelling to minimise the effects of multicollinearity, as higher VIF values indicate strong linear dependence among predictors, which can inflate the variance of estimated coefficients and compromise model stability [83].

**Figure 7:**
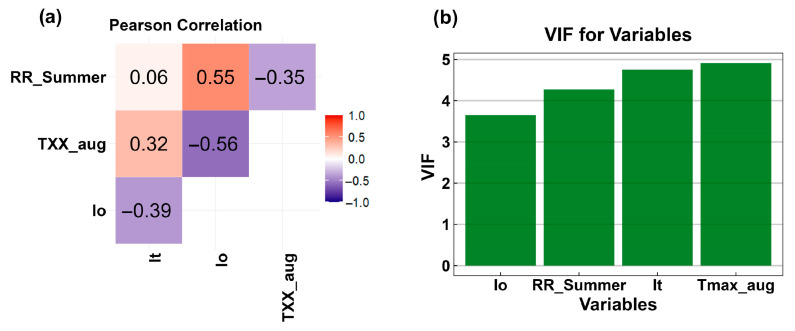
The bio-ecological indicators selected for implementation in Biomod2 met the criteria based on Pearson correlation (**a**) and variance inflation factor (VIF) (**b**) analyses.

Following these analyses, we selected the four bio-ecological indicators that met the Pearson correlation (Figure 7a), VIF analyses (Figure 7b) criteria for use in the Biomod2: Thermicity Index (It), Ombrotermic Index (Io), Accumulated summer precipitation from June to August (RR_Summer) and Maximum of the daily maximum temperature of August (TXX_aug):

1. Thermicity Index (It; dimensionless) describes the intensity of cold, which can be a restrictive factor for plants [84,85,86] (Table S8);
2. Ombrothermic Index (Io; dimensionless) is a bioclimatic index that defines the water available to vegetation to grow [84,85,86] (Table S8);
3. Accumulated summer precipitation from June to August (RR_Summer; mm): This parameter represents the total precipitation from June to August. Since marsh gentian thrives in humid environments, low summer precipitation can be a critical limiting factor for its survival and reproduction [87];
4. Maximum of the daily maximum temperature of August (TXX_aug; °C): The marsh gentian distribution is typically restricted at higher temperatures. Elevated maximum temperature, particularly in August, may negatively impact its physiological processes and limit its growth [88].

The threshold values for the thermotypes (Table S1) and ombrotypes horizons (Table S3) are defined by Rivas-Martínez [27].

### 4.5. Species Distribution Models

To create the marsh gentian distribution model, the historical climate simulation (1995–2014) variables (It, Io, RR_summer and TXX_aug) are used as predictors, and the 1277 occurrence records and pseudo-absence generated points are the predictands. Pseudo-absence points serve as surrogates of true observed absences [89]. No consensus was found in the literature for the ideal number of absences to be used, due to variation across modelling algorithms, the number of absences was selected to prioritise prevalence balance (i.e., the ratio of presences to absences) [89,90]. Five pseudo-absence datasets were generated using the random sampling method, corresponding to five prevalence ratios: 1:1 (1277), 1:3 (3831), 1:5 (6385), 1:7 (8939) and 1:9 (11493), to ensure robustness of the models [23,26]. Six modelling algorithms were selected based on their characteristics and their applications in similar studies to create the models (Table 2) [57].

The models were trained and tested, using cross-validation on an 80/20 data split. This process was repeated 10 times for each model to obtain a more stable result by avoiding model stochasticity [26,89]. Each was evaluated for its performance using five evaluation metrics (Table 3), combining non-spatial (i.e., TSS, AUCroc, BIAS, CSI) and spatial metrics (i.e., BOYCE) [22]. Effectively, most of the SDMs rely primarily on non-spatial metrics, and often fall short in accurately capturing the true geographic distribution of species [22]. In contrast, spatial metrics assess model predictions based on spatial accuracy, for example, by comparing them with geographic layers representing sampling effort or with independent data on the actual distribution of the target species [22]. These metrics were calculated in Biomod2 following the procedures outlined in the package manual [102].

Then we combined the SDMs in an ensemble model using the mean probability values (EMmean [107]) to improve the predictive accuracy, to provide reliable results and to get a more robust estimate model than the single models [26]. The EMmean output represents the unweighted average (mean) of the selected individual model predictions, grid cell by grid cell [102]. For this purpose, we selected models with a Boyce Index greater than 0.8, as values close to +1 indicate excellent model performance, while values near or below 0 suggest poor predictive ability [36,108].

To understand the variable contributions to the model and the relationships between species distribution and environmental variables, we assessed variable importance [91] and response curves [109]. The probabilistic consensus predictions are presented on a continuous scale from 0 (no suitability) to 1000 (maximum suitability). We used ArcGIS to further divide this suitability scale into four classes: very-high suitability (750–1000), high suitability (500–750), moderate suitability (250–500), and unsuitable (0–250) [36].

Finally, consensus predictions were also reclassified into presence/absence maps through BOYCE optimised thresholds available in ‘biomod2’ [102]. Suitability change was categorised into three classes based on the binary maps of presence/absence: Loss (areas currently suitable but projected to become unsuitable in the future), Stable (regions that remain suitable both now and, in the future) and Gain (currently unsuitable regions projected to become suitable) [35].

### 4.6. Evaluation of Climatic Suitability in Ecological Conservation Areas

Natura 2000 is a network of protected areas established under the European Union’s Habitats Directive (92/43/EEC) and Bird Directive (2009/147/EC), aimed at ensuring the long-term conservation of Europe’s most valuable and threatened species and habitats [45]. Similarly, the Ramsar Convention on Wetlands [7] promotes the conservation and wise use of wetlands through international cooperation and sustainable management practices.

In this study, we selected four protected areas designated under Natura 2000 (RNAP, SCI, SAC and Biosphere Reserves) and RAMSAR (Table 4), to evaluate shifts in climate suitability for *G. pneumonanthe*. We aim to assess whether current conservation designations remain effective under projected climate-driven changes in the species’ distribution.

In the historical climate simulation period, the marsh gentian suitable areas were 20.1% and 3.8% in Natura 2000 and RAMSAR, respectively. To assess changes in suitability within these protected areas, an intersection analysis was conducted between predicted changes and the designated conservation areas. Based on the results obtained, we will be able to assess the areas that will be less suitable in the future and propose measures to manage these areas to adapt to climate change.

## 5. Conclusions

As climate change progresses, the conditions across the IP are expected to become increasingly challenging, with rising temperatures and decreasing precipitation leading to the expansion of arid and semiarid climates (according to the Ombrothermic Index) and inframediterranean climates (according to the Thermicity Index). Using the biomod2 platform, which is used for the development of species distribution models, it was possible to identify the suitability areas for marsh gentian growth under historical and future scenarios. The comparison shows that many of the species’ historically suitable areas are likely to be lost. Although new suitable areas may emerge in the Pyrenees due to latitudinal and altitudinal shifts, these gains are too limited to compensate for the overall decline, representing a substantial extinction risk for *G. pneumonanthe* in the region. Currently listed as “Least Concern” on the IUCN Red List of Threatened Species, *G. pneumonanthe* L. may face a reassessment towards a higher risk category if the projected suitable area losses occur. The preservation of suitable habitats for *G. pneumonanthe* is also essential for the survival of the species that depend on it, particularly *P. alcon*. This close interdependence illustrates a cascading biodiversity threat, whereby the decline of *G. pneumonanthe* directly endangers *P. alcon*, reinforcing the urgency of proactive conservation strategies to mitigate the impacts of climate change. The study aims to support conservation entities in taking proactive action to reduce the negative impacts of climate change on ecosystems and biodiversity loss. It is also intended that these findings will inform land-use planning policies in Portugal and Spain. A coordinated approach between the two countries, combining knowledge and strategic vision, could strengthen habitat protection and species conservation. This includes identifying climate-sensitive areas, revising legislation and conservation targets, and integrating climate projections into environmental policy. For policymakers, we recommend incorporating climate projections into land-use planning, supporting active management in protected areas, and promoting monitoring programmes to track population trends. At the European level, this study may also serve as an illustrative decision-support tool that can be applied to other regions, promoting further research and guiding habitat management on wider scales.

## Figures and Tables

**Table 1:** Metric evaluation of EM according to sensitivity, specificity and calibration (dimensionless).

Evaluation Metric	Sensitivity	Specificity	Calibration
TSS	0.896	0.841	0.740
AUCroc	0.868	0.870	0.955
BOYCE	0.843	0.895	0.975
BIAS	0.591	0.982	0.936
CSI	0.498	0.991	0.411

**Table 2:** Modelling algorithms applied in the present study [91,92].

Modelling Algorithms	Abbreviation	Description
Generalized linear models [93]	GLM	GLM is a statistical regression model that assumes a linear relationship between the response variability and predictors [92].
Generalized additive models [94]	GAM	An extension of GLM, but uses nonparametric smooth functions [91].
Random forests [95]	RF	RF is an ensemble of multiple decision trees [92].
Generalised boosted models [96]	GBM	GBM is a machine learning model that sequentially combines single decision trees [97].
Classification tree analysis [98]	CTA	A simple decision tree model [99].
Multivariate adaptive regression splines [100]	MARS	Mars employs piecewise linear regression to model complex relationships [101].

**Table 3:** Modelling evaluation metrics applied in the present study.

Evaluation Metrics	Abbreviation	Description
Area under the receiver operating characteristic curve [103]	AUCroc	Measures the model’s ability to distinguish between presence and absence [103].
Bias score (frequency bias) [104]	BIAS	Measures the ratio of the frequency of predicted events to observed events [104].
Boyce Index [105]	BOYCE	Evaluates the performance of presence-only models within the known presence-only [106].
Critical Success Index (threat score) [104]	CSI	Indicates the fraction of correctly predicted presences (hits) out of all actual and predicted presences [104].

**Table 4:** Natura 2000 and RAMSAR conservation areas.

Directive	Designation of Area	Database	Collection
Natura 2000 Network [45]	Rede Nacional de Áreas Protegidas (RNAP)	[110]	Natura 2000
	Sites of Community Importance (SCI)	[111,112]	
	Special Areas of Conservation (SAC)		
Man and the Biosphere Programme (MAB)	Biosphere Reserves	[113,114]	
Convention on Wetlands [7]	Wetlands of International Importance (RAMSAR)	[115,116]	RAMSAR

## Data Availability

The data supporting the results are available in a public repository at GBIF.org: GBIF Occurrence Download https://doi.org/10.15468/dl.9kxash (accessed on 30 December 2024) and GBIF Occurrence Download https://doi.org/10.15468/dl.mjckf5 (accessed on 16 April 2025).

## Supplementary Materials

The following supporting information can be downloaded at: https://www.mdpi.com/article/10.3390/plants14182857/s1, Table S1. Thermotypes and respective lower and upper horizons thresholds [27], Table S2. *G. pneumonanthe* occurrence percentages (%) across the classes of each bioclimatic index considered: Thermicity Index (It), Ombrothermic Index (Io), Accumulated Summer Precipitation from June to August (RR_summer), and Maximum of the Daily Maximum Temperature of August (TXX_aug). Table S3. Ombrotypes and respective lower and upper horizons thresholds [27], Table S4. Metric evaluation by pseudo-absences, metric evaluation and algorithm methods, Table S5. Data collection with occurrence locations from six databases, between 1851 and 2024, Table S6. Description of the Global Climate Models (GCMs) applied in the present study, Table S7. Bi-ecological indicators (14 bioclimatic indices and 2 topographic indicators) and respective abbreviations and units, Table S8. Thermicity and Ombrothermic Indices according to Rivas-Martínez [27], Figure S1. Ensemble means bio-ecological indicators for future scenarios: SSP5-8.5 (2041–2060) and SSP3-7.0 (2081–2100). The maps show: Thermicity index (It; a, b), Ombrothermic index (Io; c, d), Accumulated summer precipitation (June–August) in mm (RR_summer; e,f), and Maximum of the daily maximum temperature of the hottest month (August) in °C (TXX_aug; g, h). The threshold values for the thermotypes and ombrotypes horizons are defined by Rivas-Martínez [27], Figure S2. Calibration (a), validation (b) by algorithms: CTA, GAM, GBM, GLM, MARS and RF, Figure S3. Bio-ecological indicators importance: Io (a,e), It (b,f), RR_summer (c,g) and TXX_aug (d,h), according to each algorithm and model ensemble, respectively, Figure S4. Response curves between bio-ecological indicators, It (a), Io (b), RR_summer (c) and TXX_aug (d), and the presence probability, in the model ensemble, Figure S5. Climatic suitability classes (high, considerable, moderate and unsuitable) and binary distribution (1 = presence and 0 = absence) are represented for future scenarios: SSP5-8.5 for 2041–2060 (a,c) and SSP3-7.0 for 2081–2100 (e,g). The corresponding area percentages for a suitable habitat, based on suitability classes and binary classification, are presented for SSP5-8.5 (b,d) and SSP3-7.0 (f,h), Figure S6. Dynamic changes in suitable areas between historical and future periods are illustrated. The changes are represented according to three categories: loss, stable and gain. These spatial distributions are represented in the maps, while their area percentage are presented in the graphs. Panels (a) and (b) correspond to SSP5-8.5 for 2041–2060, and panels (c) and (d) correspond to SSP3-7.0 for 2081–2100, Figure S7. Natura 2000 and RAMSAR distribution in the Iberian Peninsula (a). Quantification of changes in suitability within Natura 2000 (b, d) and RAMSAR (c,e) areas, from the historical (1995–2014) period to future scenarios: SSP5-8.5 (2041–2060) and SSP3-7.0 (2081–2100). Figure S8. Pearson correlation for the 16 bio-ecological variables considered in the present study.

## References

[1] Popović Z., Krstić-Milošević D., Stefanović M., Matić R., Vidaković V., Bojović S. (2019). Chemical and Morphological Inter- and Intrapopulation Variability in Natural Populations of *Gentiana pneumonanthe* L. *Chem*. Biodivers..

[2] Daoud-Bouattour A., Gammar-Ghrabi Z., Limam-Ben Saad S., Muller S.D. The IUCN Red List of Threatened Species 2010—*Gentiana pneumonanthe* (Mediterranean Assessment). https://www.iucnredlist.org/species/164379/5849081.

[3] Ben Saad S., Bilz M., Daoud-Bouattour A., Ghrabi Z., Muller S. The IUCN Red List of Threatened Species 2012—*Gentiana pneumonanthe* (Europe Assessment). https://www.iucnredlist.org/species/164379/1047003#geographic-range.

[4] Oostermeijer J., Altenburg R.M., Den Nijs H. (1995). Effects of Outcrossing Distance and Selling on Fitness 741 Components in the Rare *Gentiana pneumonanthe* (Gentianaceae). Acta Botanica Neerlandica.

[5] Fonseca A., Santos J., Pádua L., Santos M. (2023). Unveiling the Future of Relict Mediterranean Mountain Peatlands by Integrating the Potential Response of Ecological Indicators with Environmental Suitability Assessments. Ecol. Indic..

[6] Pierce S., Spada A., Caporali E., Puglisi F., Panzeri A., Luzzaro A., Cislaghi S., Mantegazza L., Cardarelli E., Labra M. (2018). Identifying Population Thresholds for Flowering Plant Reproductive Success: The Marsh Gentian (*Gentiana pneumonanthe*) as a Flagship Species of Humid Meadows and Heathland. Biodivers. Conserv..

[7] Ramsar Ramsar. https://www.ramsar.org/.

[8] EEA Natura 2000. https://www.eea.europa.eu/en/datahub/datahubitem-view/6fc8ad2d-195d-40f4-bdec-576e7d1268e4.

[9] Warson J., Baguette M., Stevens V.M., Honnay O., De Kort H. (2023). The Impact of Habitat Loss on Molecular Signatures of Coevolution between an Iconic Butterfly (Alcon Blue) and Its Host Plant (Marsh Gentian). J. Hered..

[10] Valdés A., Ehrlén J. (2022). Microclimate Influences Plant Reproductive Performance via an Antagonistic Interaction. Basic Appl. Ecol..

[11] Cormont A., Wieger Wamelink G.W., Jochem R., WallisDeVries M.F., Wegman R.M.A. (2013). Host Plant-Mediated Effects of Climate Change on the Occurrence of the Alcon Blue Butterfly (*Phengaris alcon*). Ecol. Modell..

[12] Rose R.J., Clarke R.T., Chapman S.B. (1998). Individual Variation and the Effects of Weather, Age and Flowering History on Survival and Flowering of the Long-Lived Perennial *Gentiana pneumonanthe*. Ecography.

[13] De Kort H., Prunier J.G., Tessier M., Turlure C., Baguette M., Stevens V.M. (2018). Interacting Grassland Species under Threat of Multiple Global Change Drivers. J. Biogeogr..

[14] He X., Liang J., Zeng G., Yuan Y., Li X. (2019). The Effects of Interaction between Climate Change and Land-Use/Cover Change on Biodiversity-Related Ecosystem Services. Glob. Chall..

[15] Krzosek K., Nowicki P. (2025). Quantification of Land Use Threats to a Flagship Species and Its Meadow Habitats within Urban Landscape. Eur. Zool. J..

[16] Freitas T.R., Santos J.A., Paredes P., Fraga H. (2024). Future Aridity and Drought Risk for Traditional and Super-Intensive Olive Orchards in Portugal. Clim. Change.

[17] Claro A.M., Fonseca A., Fraga H., Santos J.A. (2023). Susceptibility of Iberia to Extreme Precipitation and Aridity: A New High-Resolution Analysis over an Extended Historical Period. Water.

[18] Riahi K., van Vuuren D.P., Kriegler E., Edmonds J., O’Neill B.C., Fujimori S., Bauer N., Calvin K., Dellink R., Fricko O. (2017). The Shared Socioeconomic Pathways and Their Energy, Land Use, and Greenhouse Gas Emissions Implications: An Overview. Glob. Environ. Change.

[19] Adhikari B., Subedi S.C., Bhandari S., Baral K., Lamichhane S., Maraseni T. (2023). Climate-Driven Decline in the Habitat of the Endemic Spiny Babbler (*Turdoides nipalensis*). Ecosphere.

[20] Fernandes A., Kovač N., Fraga H., Fonseca A., Šućur Radonjić S., Simeunović M., Ratković K., Menz C., Costafreda-Aumedes S., Santos J.A. (2024). Challenges to Viticulture in Montenegro under Climate Change. ISPRS Int. J. Geoinf..

[21] Jia T., Qi Y., Zhao H., Xian X., Li J., Huang H., Yu W., Liu W.X. (2023). Estimation of Climate-Induced Increased Risk of *Centaurea solstitialis* L. Invasion in China: An Integrated Study Based on Biomod2. Front. Ecol. Evol..

[22] Bracho-Estévanez C.A., Arenas-Castro S., González-Varo J.P., González-Moreno P. (2024). Spatially Explicit Metrics Improve the Evaluation of Species Distribution Models Facing Sampling Biases. Ecol. Inform..

[23] Guo L., Gao Y., He P., He Y., Meng F. (2023). Modeling for Predicting the Potential Geographical Distribution of Three Ephedra Herbs in China. Plants.

[24] Sofaer H.R., Jarnevich C.S., Pearse I.S., Smyth R.L., Auer S., Cook G.L., Edwards T.C., Guala G.F., Howard T.G., Morisette J.T. (2019). Development and Delivery of Species Distribution Models to Inform Decision-Making. Bioscience.

[25] Breiner F.T., Guisan A., Bergamini A., Nobis M.P. (2015). Overcoming Limitations of Modelling Rare Species by Using Ensembles of Small Models. Methods Ecol. Evol..

[26] Adão F., Campos J.C., Santos J.A., Malheiro A.C., Fraga H. (2023). Relocation of Bioclimatic Suitability of Portuguese Grapevine Varieties under Climate Change Scenarios. Front. Plant Sci..

[27] Rivas-Martínez S., Rivas Sáenz S., Penas A., Alcaraz F., Amigo J., Asensi A., Barbour M., Biondi E., Cantó P., Capelo J. (2011). Worldwide Bioclimatic Classification System. Glob. Geobot..

[28] Costa R., Fraga H., Fernandes P.M., Santos J.A. (2017). Implications of Future Bioclimatic Shifts on Portuguese Forests. Reg. Environ. Change.

[29] Andrade C., Fonseca A., Santos J.A. (2021). Are Land Use Options in Viticulture and Oliviculture in Agreement with Bioclimatic Shifts in Portugal?. Land.

[30] Araújo P.V., Portela-Pereira E., Lourenço J., Clemente A., Clamote F., Almeida J.D., Pereira A.J., Porto M. *Gentiana pneumonanthe* L.—Mapa de Distribuição. http://www.flora-on.pt/#wGentiana+pneumonanthe.

[31] Cuena-Lombraña A., Porceddu M., Dettori C.A., Bacchetta G. (2020). Predicting the Consequences of Global Warming on *Gentiana lutea* Germination at the Edge of Its Distributional and Ecological Range. PeerJ.

[32] Quinta L. A Borboleta Da Serra Do Alvão Que Parece Retirada de Uma Fábula. https://www.nationalgeographic.pt/meio-ambiente/a-borboleta-da-serra-do-alvao-que-parece-retirada-uma-fabula_1245.

[33] Kanda R.Z., Da S.S., Maârouhi I.M., Issoufou A.A., Ouattara D. (2024). Assessment of Climate Change Impact on Future Distribution of Palm Trees in Niger, West Africa. Discov. Sustain..

[34] Karger D.N., Wilson A.M., Mahony C., Zimmermann N.E., Jetz W. (2021). Global Daily 1 Km Land Surface Precipitation Based on Cloud Cover-Informed Downscaling. Sci. Data.

[35] Wang Z., Zhuo Z., Liu B., Peng Y., Xu D. (2025). Predicting the Future Geographic Distribution of the Traditional Chinese Medicinal Plant *Epimedium acuminatum* Franch. in China Using Ensemble Models Based on Biomod2. Plants.

[36] Biancolini D., Pacifici M., Falaschi M., Bellard C., Blackburn T.M., Ficetola G.F., Rondinini C. (2024). Global Distribution of Alien Mammals Under Climate Change. Glob. Change Biol..

[37] Campos J.C., Albuquerque B., Civantos E., Honrado J.P., Regos A. (2025). Unveiling the Effects of Landscape–Fire Interactions on Functional Diversity in a Southern European Mountain. Ecol. Appl..

[38] Hu H., Wei Y., Wang W., Wang C. (2021). The Influence of Climate Change on Three Dominant Alpine Species under Different Scenarios on the Qinghai–Tibetan Plateau. Diversity.

[39] Barbosa W.L., Alves-Souza S.N. (2025). Data Quality Issues in Data Used in Species Distribution Models: A Systematic Literature Review. Ecol. Inform..

[40] Fiorentino D., Núñez-Riboni I., Pierce M.E., Oesterwind D., Akimova A. (2025). Improving Species Distribution Models for Climate Change Studies: Ecological Plausibility and Performance Metrics. Ecol. Modell..

[41] Lienert J., Diemer M., Schmid B. (2002). Effects of Habitat Fragmentation on Population Structure and Fitness Components of the Wetland Specialist *Swertia perennis* L. (Gentianaceae) Basic and Applied Ecology. Basic Appl. Ecol..

[42] Petanidou T., Ellis-Adam A.C., Den H.C.M., Gerard J., Oostermeijer B. (2001). Differential Pollination Success in the Course of Individual Flower Development and Flowering Time in *Gentiana pneumonanthe* L. (Gentianaceae). Bot. J. Linn. Soc..

[43] Soares P.O., Crespi A.L., Rodrigues M.C., Arnaldo P.S. (2012). The Habitat Vegetational Structure and the Success of the Blue Alcon, *Maculinea alcon* (Denis & Schiffermüller). Plant Biosyst..

[44] Surrey Wildlife Trust Marsh Gentian. https://www.surreywildlifetrust.org/marsh-gentian.

[45] European Commission The Habitats Directive. https://environment.ec.europa.eu/topics/nature-and-biodiversity/habitats-directive_en.

[46] European Parliament of the European Union; Council of the European Union Regulation (EU) 2024/1991 of the European Parliament and of the Council of 24 June 2024 on Nature Restoration and Amending Regulation (EU) 2022/869. https://eur-lex.europa.eu/eli/reg/2024/1991/oj/eng.

[47] European Commission; Directorate-General for Environment EU Biodiversity Strategy for 2030. https://eur-lex.europa.eu/legal-content/EN/TXT/?uri=celex%3A52020DC0380.

[48] Oostermeijer J.G.B., Den Nijs J.C.M., Raijmann L.E.L., Menken S.B.J. (1992). Population Biology and Management of the Marsh Gentian (*Gentiana pneumonanthe* L.), a Rare Species in The Netherlands. Bot. J. Linn. Soc..

[49] Catorci A., Cesaretti S., Malatesta L., Tardella F.M. (2014). Effects of Grazing vs Mowing on the Functional Diversity of Sub-Mediterranean Productive Grasslands. Appl. Veg. Sci..

[50] Maes D., Pardon W., Palmans G., Van Dyck H. (2024). The Last of the Maculineans: Can We Save the Emblematic Alcon Blue Butterfly *Phengaris alcon* under Climate Change When Its Habitat Continues to Deteriorate?. J. Insect. Conserv..

[51] Moschetti M., Besnard A., Couturier T., Fonderflick J. (2020). Grazing Intensity Negatively Affects the Maintenance of *Gentiana pneumonanthe* and the Survival of *Phengaris alcon* Egg-Laying. J. Insect. Conserv..

[52] Freitas T.R., Santos J.A., Silva A.P., Fonseca A., Fraga H. (2023). Evaluation of Historical and Future Thermal Conditions for Almond Trees in North—Eastern Portugal. Clim. Change.

[53] Raijmann L.E.L., Van Leeuwen N.C., Kersten R., Oostermeijer J.G.B., Den Nijs H.C.M., Menken S.B.J. (1994). Genetic Variation and Outcrossing Rate in Relation to Population Size in *Gentiana pneumonanthe* L. *Conserv*. Biol..

[54] Anthos Listados de Gentiana Pneumonanthe (Fam. Gentianaceae) y Táxones Infraespecíficos. http://www.anthos.es/.

[55] GBIF.org GBIF Occurrence Download. https://www.gbif.org/occurrence/download/0051735-241126133413365.

[56] iNaturalist.org INaturalist-Gentiana Pneumonanthe. https://www.inaturalist.org/observations/export?flow_task_id=516049.

[57] GBIF.org GBIF Occurrence Download. https://www.gbif.org/occurrence/download/0002669-250415084134356.

[58] Huang D., An Q., Huang S., Tan G., Quan H., Chen Y., Zhou J., Liao H. (2023). Biomod2 Modeling for Predicting the Potential Ecological Distribution of Three Fritillaria Species under Climate Change. Sci. Rep..

[59] Aiello-Lammens M.E., Boria R.A., Radosavljevic A., Vilela B., Anderson R.P. (2015). SpThin: An R Package for Spatial Thinning of Species Occurrence Records for Use in Ecological Niche Models. Ecography.

[60] Boria R.A., Olson L.E., Goodman S.M., Anderson R.P. (2014). Spatial Filtering to Reduce Sampling Bias Can Improve the Performance of Ecological Niche Models. Ecol. Modell..

[61] Swart N.C., Cole J.N.S., Kharin V.V., Lazare M., Scinocca J.F., Gillett N.P., Anstey J., Arora V., Christian J.R., Hanna S. (2019). The Canadian Earth System Model Version 5 (CanESM5.0.3). Geosci. Model Dev..

[62] Voldoire A., Saint-Martin D., Sénési S., Decharme B., Alias A., Chevallier M., Colin J., Guérémy J.F., Michou M., Moine M.P. (2019). Evaluation of CMIP6 DECK Experiments With CNRM-CM6-1. J. Adv. Model. Earth Syst..

[63] Séférian R., Nabat P., Michou M., Saint-Martin D., Voldoire A., Colin J., Decharme B., Delire C., Berthet S., Chevallier M. (2019). Evaluation of CNRM Earth System Model, CNRM-ESM2-1: Role of Earth System Processes in Present-Day and Future Climate. J. Adv. Model. Earth Syst..

[64] Döscher R., Acosta M., Alessandri A., Anthoni P., Arsouze T., Bergman T., Bernardello R., Boussetta S., Caron L.P., Carver G. (2022). The EC-Earth3 Earth System Model for the Coupled Model Intercomparison Project 6. Geosci. Model Dev..

[65] Lurton T., Balkanski Y., Bastrikov V., Bekki S., Bopp L., Braconnot P., Brockmann P., Cadule P., Contoux C., Cozic A. (2020). Implementation of the CMIP6 Forcing Data in the IPSL-CM6A-LR Model. J. Adv. Model. Earth Syst..

[66] Tatebe H., Ogura T., Nitta T., Komuro Y., Ogochi K., Takemura T., Sudo K., Sekiguchi M., Abe M., Saito F. (2019). Description and Basic Evaluation of Simulated Mean State, Internal Variability, and Climate Sensitivity in MIROC6. Geosci. Model Dev..

[67] Gutjahr O., Putrasahan D., Lohmann K., Jungclaus J.H., Von Storch J.S., Brüggemann N., Haak H., Stössel A. (2019). Max Planck Institute Earth System Model (MPI-ESM1.2) for the High-Resolution Model Intercomparison Project (HighResMIP). Geosci. Model Dev..

[68] Yukimoto S., Kawai H., Koshiro T., Oshima N., Yoshida K., Urakawa S., Tsujino H., Deushi M., Tanaka T., Hosaka M. (2019). The Meteorological Research Institute Earth System Model Version 2.0, MRI-ESM2.0: Description and Basic Evaluation of the Physical Component. J. Meteorol. Soc. Jpn..

[69] Sellar A.A., Jones C.G., Mulcahy J.P., Tang Y., Yool A., Wiltshire A., O’Connor F.M., Stringer M., Hill R., Palmieri J. (2019). UKESM1: Description and Evaluation of the U.K. Earth System Model. J. Adv. Model. Earth Syst..

[70] Karger D.N., Conrad O., Böhner J., Kawohl T., Kreft H., Soria-Auza R.W., Zimmermann N.E., Linder H.P., Kessler M. (2017). Climatologies at High Resolution for the Earth’s Land Surface Areas. Sci. Data.

[71] Frieler K., Volkholz J., Lange S., Schewe J., Mengel M., Del Rocío Rivas López M., Otto C., Reyer C.P.O., Karger D.N., Malle J.T. (2024). Scenario Setup and Forcing Data for Impact Model Evaluation and Impact Attribution within the Third Round of the Inter-Sectoral Impact Model Intercomparison Project (ISIMIP3a). Geosci. Model Dev..

[72] Lange S. (2021). ISIMIP3b Bias Adjustment Fact Sheet.

[73] Lange S. ISIMIP3BASD (2.5.0). https://zenodo.org/records/4686991.

[74] Lange S. (2019). Trend-Preserving Bias Adjustment and Statistical Downscaling with ISIMIP3BASD (v1.0). Geosci. Model Dev..

[75] Eyring V., Bony S., Meehl G.A., Senior C.A., Stevens B., Stouffer R.J., Taylor K.E. (2016). Overview of the Coupled Model Intercomparison Project Phase 6 (CMIP6) Experimental Design and Organization. Geosci. Model Dev..

[76] O’Neill B.C., Tebaldi C., Van Vuuren D.P., Eyring V., Friedlingstein P., Hurtt G., Knutti R., Kriegler E., Lamarque J.F., Lowe J. (2016). The Scenario Model Intercomparison Project (ScenarioMIP) for CMIP6. Geosci. Model Dev..

[77] Brun P., Zimmermann N.E., Hari C., Pellissier L., Karger D.N. (2022). Global Climate-Related Predictors at Kilometer Resolution for the Past and Future. Earth Syst. Sci. Data.

[78] Martinez A., Iglesias G. (2021). Wind Resource Evolution in Europe under Different Scenarios of Climate Change Characterised by the Novel Shared Socioeconomic Pathways. Energy Convers. Manag..

[79] Freitas T.R., Santos J.A., Fernandes A., Menz C., Paredes P., Fraga H. (2025). Future Agroclimatic Suitability for Oliviculture in Portugal Based on a New High-Resolution Climate Dataset. Mitig. Adapt. Strateg. Glob. Change.

[80] Mesquita S., Sousa A.J. (2009). Bioclimatic Mapping Using Geostatistical Approaches: Application to Mainland Portugal. Int. J. Climatol..

[81] Loidi J., Navarro-Sánche G., Vynokurov D. (2022). Climatic Definitions of the World’s Terrestrial Biomes. Veg. Classif. Surv..

[82] Fonseca A.R., Santos J.A. (2018). High-Resolution Temperature Datasets in Portugal from a Geostatistical Approach: Variability and Extremes. J. Appl. Meteorol. Climatol..

[83] Andrade C., Contente J., Santos J.A. (2021). Climate Change Projections of Aridity Conditions in the Iberian Peninsula. Water.

[84] Akinwande M.O., Dikko H.G., Samson A. (2015). Variance Inflation Factor: As a Condition for the Inclusion of Suppressor Variable(s) in Regression Analysis. Open J. Stat..

[85] Gopar-Merino L.F., Velazquez A., De Azcarate J.G. (2015). Bioclimatic Mapping as a New Method to Assess Effects of Climatic Change. Ecosphere.

[86] Pesaresi S., Galdenzi D., Biondi E., Casavecchia S. (2014). Bioclimate of Italy: Application of the Worldwide Bioclimatic Classification. J. Maps.

[87] Monteiro-Henriques T., Martins M.J., Cerdeira J.O., Silva P., Arsénio P., Silva A., Bellu A., Costa J.C. (2016). Bioclimatological Mapping Tackling Uncertainty Propagation: Application to Mainland Portugal. Int. J. Climatol..

[88] Pradhan D.K., Cahalan C., Ulak S. (2018). Effects of Predicted Reduced Summer Rainfall on Growth and Development of Silver Birch (Betula Pendula Roth) and Downy Birch (Betula Pubescens Ehrh). For. J. Inst. For..

[89] Hatfield J.L., Prueger J.H. (2015). Temperature Extremes: Effect on Plant Growth and Development. Weather Clim. Extrem..

[90] Barbet-Massin M., Jiguet F., Albert C.H., Thuiller W. (2012). Selecting Pseudo-Absences for Species Distribution Models: How, Where and How Many?. Methods Ecol. Evol..

[91] Santini L., Benítez-López A., Maiorano L., Čengić M., Huijbregts M.A.J. (2021). Assessing the Reliability of Species Distribution Projections in Climate Change Research. Divers. Distrib..

[92] Valavi R., Guillera-Arroita G., Lahoz-Monfort J.J., Elith J. (2022). Predictive Performance of Presence-Only Species Distribution Models: A Benchmark Study with Reproducible Code. Ecol. Monogr..

[93] Hao T., Elith J., Guillera-Arroita G., Lahoz-Monfort J.J. (2019). A Review of Evidence about Use and Performance of Species Distribution Modelling Ensembles like BIOMOD. Divers. Distrib..

[94] McCullagh P. (1984). Generalized Linear Models. Eur. J. Oper. Res..

[95] Hastie T., Tibshirani R. (1986). Generalized Additive Models. Stat. Sci..

[96] Breiman L. (2001). Random Forests. Mach. Learn..

[97] Ridgeway G. (1999). The State of Boosting.

[98] Konstantinov A.V., Utkin L.V. (2021). Interpretable Machine Learning with an Ensemble of Gradient Boosting Machines. Knowl. Based Syst..

[99] Breiman L., Friedman J.H., Olshen R.A., Stone C.J. (2017). Classification and Regression Trees.

[100] Breiman L., Friedman J.H., Olshen R.A., Stone C.J. (1984). Classification and Regression Trees.

[101] Friedman J.H. (1991). Multivariate Adaptive Regression Splines. Ann. Stat..

[102] Boehmke B., Greenwell B. Hands-On Machine Learning with R. https://bradleyboehmke.github.io/HOML/.

[103] Thuiller W., Lafourcade B., Engler R., Araújo M.B. (2009). BIOMOD—A Platform for Ensemble Forecasting of Species Distributions. Ecography.

[104] Hanley J.A., McNeil B.J. (1982). The Meaning and Use of the Area under a Receiver Operating Characteristic (ROC) Curve. Radiology.

[105] CAWCR WWRP/WGNE Joint Working Group on Forecast Verification Research. https://community.wmo.int/en/activity-areas/wwrp/wwrp-working-groups/wwrp-forecast-verification-research.

[106] Hirzel A.H., Le Lay G., Helfer V., Randin C., Guisan A. (2006). Evaluating the Ability of Habitat Suitability Models to Predict Species Presences. Ecol. Modell..

[107] Somodi I., Bede-Fazekas Á., Botta-Dukát Z., Molnár Z. (2024). Confidence and Consistency in Discrimination: A New Family of Evaluation Metrics for Potential Distribution Models. Ecol. Modell..

[108] Guéguen M., Blancheteau H., Thuiller W. Biomod2: Ensemble Platform for Species Distribution Modeling 2025. https://cran.r-project.org/web/packages/biomod2/biomod2.pdf.

[109] Patiño J., Collart F., Vanderpoorten A., Martin-Esquivel J.L., Naranjo-Cigala A., Mirolo S., Karger D.N. (2023). Spatial Resolution Impacts Projected Plant Responses to Climate Change on Topographically Complex Islands. Divers. Distrib..

[110] Elith J., Ferrier S., Huettmann F., Leathwick J. (2005). The Evaluation Strip: A New and Robust Method for Plotting Predicted Responses from Species Distribution Models. Ecol. Modell..

[111] ICNF Áreas Protegidas Que Integram a Rede Nacional de Áreas Protegidas. https://www.icnf.pt/conservacao/rnapareasprotegidas.

[112] ICNF Rede Natura 2000. https://www.icnf.pt/conservacao/redenatura2000.

[113] MITECO Red Natura 2000. https://www.miteco.gob.es/es/biodiversidad/temas/espacios-protegidos/red-natura-2000.html.

[114] MITECO Reservas de La Biosfera. https://www.miteco.gob.es/es/biodiversidad/servicios/banco-datos-naturaleza/informacion-disponible/mab_descargas.html.

[115] ICNF Informação Geográfica. https://geocatalogo.icnf.pt/catalogo_tema1.html.

[116] MITECO Lista Ramsar y Aportación Española. https://www.miteco.gob.es/es/biodiversidad/temas/ecosistemas-y-conectividad/conservacion-de-humedales/ch_hum_ramsar_esp_lista.html.

