# Supplementary material for: Bio-Ecological Indicators for Gentiana pneumonanthe L. Climatic Suitability in the Iberian Peninsula
Source: Plants (Basel). 2025 Sep 12;14(18):2857. doi: 10.3390/plants14182857 (PMC12473960; doi:10.3390/plants14182857)
Supplement: Supplementary file 1 [file plants-14-02857-s001.zip › Supplementar_material.pdf]

# Bio-ecological indicators for *Gentiana pneumonanthe* L. climatic suitability in the Iberian Peninsula

**Teresa. R. Freitas [1*], Sílvia Martins [1,2], Joaquim Jesus [1,2], João Campos [3,4], António Fernandes [1], Christoph Menz [5], Ernestino Maravalhas [6], Helder Fraga [1], João A. Santos [1]**

[1] Centre for the Research and Technology of Agroenvironmental and Biological Sciences, CITAB, Inov4Agro, University of Trás-os-Montes e Alto Douro, UTAD, Quinta de Prados, 5000–801 Vila Real, Portugal; A.F.: acpf91@utad.pt, H.F.: hfraga@utad.pt, J.A.S.: jsantos@utad.pt
[2] Laboratory of Fluvial and Terrestrial Ecology, LEFT, University of Trás-os-Montes e Alto Douro, UTAD, Quinta de Prados, 5000–801 Vila Real, Portugal; S.M.: silviamartins@utad.pt, J.J.: jjesus@utad.pt
[3] Centro de Investigação em Biodiversidade e Recursos Genéticos, CIBIO, InBIO Laboratório Associado, Universidade do Porto, Campus de Vairão, 4485–661 Vairão, Portugal.; J.C.: jc_campos@cibio.up.pt
[4] BIOPOLIS Program in Genomics, Biodiversity and Land Planning, CIBIO, Campus de Vairão, 4485–661 Vairão, Portugal; J.C.: jc_campos@cibio.up.pt
[5] Potsdam Institute for Climate Impact Research e. V., PIK, Telegrafenberg A 31, 14473 Potsdam, Germany; C.M.: christoph.menz@pik-potsdam.de
[6] Centro de Conservação das Borboletas de Portugal, TAGIS, Rua das Portas de Évora, 3, 7480–152, Avis, Portugal; E.M.: emsmaravalhas@gmail.com
* Correspondence: trfreitas@utad.pt

**Table S1.** Thermotypes and respective lower and upper horizons thresholds [27].

| Thermotypes horizons | It (dimensionless) |
|---|---|
| Inframediterranean Upper | 451 – 515 |
| Thermomediterranean Lower | 401 – 450 |
| Thermomediterranean Upper | 351 – 400 |
| Mesomediterranean Lower | 286 – 350 |
| Mesomediterranean Upper | 221 – 285 |
| Supramediterranean Lower | 151 – 220 |
| Supramediterranean Upper | <= 150 |

**Table S2.** *G. pneumonanthe* occurrence percentages (%) across the classes of each bioclimatic index considered: Thermicity Index (It), Ombrothermic Index (Io), Accumulated Summer Precipitation from June to August (RR_summer), and Maximum of the Daily Maximum Temperature of August (TXX_aug).

| Index | Class | *G. pneumonanthe* occurrences (%) |
|---|---|---|
| It (dimensionless) | 515 < | 0.0 |
| | 450 – 515 | 0.1 |
| | 400 – 450 | 6.9 |
| | 350 – 400 | 15.9 |
| | 285 – 350 | 26.8 |
| | 220 – 285 | 28.7 |
| | 150 – 200 | 17.1 |
| | 50 – 150 | 4.0 |
| | -50 – -50 | 0.5 |
| | -155 – -50 | 0.0 |
| Io (dimensionless) | 17.0 – 18.0 | 0.0 |
| | 12.0 – 17.0 | 1.3 |
| | 8.5 – 12.0 | 8.0 |
| | 6.0 – 8.5 | 10.8 |
| | 4.6 – 6.0 | 10.9 |
| | 3.6 – 4.6 | 19.6 |
| | 2.7 – 3.6 | 31.8 |
| | 2.0 – 2.7 | 14.0 |
| | 1.4 – 2.0 | 2.8 |
| | 1.0 – 1.4 | 0.9 |
| | 0.6 – 1.0 | 0.0 |
| | 0.4 – 0.6 | 0.0 |
| RR_Summer (mm) | 230 – 250 | 0.0 |
| | 210 – 230 | 0.4 |
| | 190 – 210 | 2.7 |
| | 170 – 190 | 4.3 |
| | 150 – 170 | 16.8 |
| | 130 – 150 | 24.7 |
| | 110 – 130 | 11.3 |
| | 90 – 110 | 17.0 |
| | 70 – 90 | 12.1 |
| | 50 – 70 | 6.3 |
| | 30 – 50 | 3.6 |
| | 10 – 30 | 0.7 |
| TXX_aug (°C) | 47 – 50 | 0.0 |
| | 44 – 47 | 0.0 |

| | |
|---|---|
| 41 – 44 | 0.0 |
| 38 – 41 | 0.3 |
| 35 – 38 | 5.1 |
| 32 – 35 | 28.3 |
| 29 – 32 | 53.5 |
| 26 – 29 | 12.4 |
| 23 – 26 | 0.3 |
| 20 – 23 | 0.0 |

**Table S3.** Ombrotypes and respective lower and upper horizons thresholds [27].

| Ombrotypes horizons | Io (dimensionless) |
|---|---|
| Arid lower | 0.4 – 0.6 |
| Arid Upper | 0.6 – 1.0 |
| Semiarid lower | 1.0 – 1.4 |
| Semiarid Upper | 1.4 – 2.0 |
| Dry lower | 2.0 – 2.7 |
| Dry Upper | 2.7 – 3.6 |
| Subhumid lower | 3.6 – 4.6 |
| Subhumid Upper | 4.6 – 6.0 |
| Humid lower | 6.0 – 8.5 |
| Humid Upper | 8.5 – 12.0 |
| Hyperhumid lower | 12.0 – 17.0 |
| Hyperhumid Upper | 17.0 – 24.0 |

**Table S4.** Metric evaluation by pseudo-absences, metric evaluation and algorithm methods.

(Presented in an Excel file)

**Table S5.** Data collection from six databases, spanning the period from 1851 to 2024, with occurrence locations.

| Occurrence number | Collection period | Database | Reference |
|---|---|---|---|
| 458 | 1851–2024 | *Anthos.es* | [54] |
| 39 | 2001–2024 | *Flora.on* | [30] |
| 2000 | 2001–2024 | *GBIF* | [55,57] |
| 116 | 2018–2024 | iNaturalist | [56] |
| 3 | 2024 | Collected by the Tâmega Electroproduction System (SET) project. | |
| 106 | 1983–2024 | The author, Ernestino Maravalhas, provided the database. | |

**Table S6.** Description of the Global Climate Models (GCMs) applied in the present study.

| Global Climate Model (GCM) | Abbreviation |
|---|---|
| Canadian Earth System Model version 5 [61] | CanESM5 |
| Centre National de Recherches Météorologiques - Climate Model Version 6.1 [62] | CNRM-CM6-1 |
| CNRM-ESM2-1 stands for the Centre National de Recherches Météorologiques (CNRM) Earth System Model, version 2-1 [63] | CNRM-ESM2-1 |
| European Community Earth System Model, version 3 [64] | EC-Earth3 |

| | |
|---|---|
| Institut Pierre-Simon Laplace Earth System Model—Coupled Model version 6A—Low Resolution [65] | IPSL-CM6A-LR |
| Model for Interdisciplinary Research on Climate, version 6 [66] | MIROC6 |
| Max Planck Institute Earth System Model Version 1.2—High Resolution [67] | MPI-ESM1-2-HR |
| Meteorological Research Institute Earth System Model Version 2.0 [68] | MRI-ESM2-0 |
| UK Earth System Model 1.0 Low Resolution [69] | UKESM1-0-LL |

**Table S7.** Bi-ecological indicators (14 bioclimatic indices and 2 topographic indicators) and respective abbreviations and units.

| Bio-ecological indicator designations | Abbreviations (units) |
|---|---|
| Thermicity Index | It (dimensionless) |
| Simple Continentality Index | Ic (°C) |
| Annual Ombrotermic Index | Io (dimensionless) |
| Maximum of the daily maximum temperature of August | TXX_aug (°C) |
| Minimum of the daily minimum temperature of January | TNN_Jan (°C) |
| Mean maximum temperature in summer (June to August) | Tmax_summer (°C) |
| Daily mean temperature | TmeanD (°C) |
| Annual accumulated precipitation | RR_annual (mm) |
| Accumulated winter precipitation (December to February) | RR_Winter (mm) |
| Accumulated summer precipitation (June to August) | RR_Summer (mm) |
| Accumulated precipitation during the growing and flowering seasons (March to October) | RR_mar_oct (mm) |
| Precipitation percent due to R95p days | R95PTOT (%) |
| Very warm days percent w.r.t. 90th percentile of the reference period | TX90P (%) |
| De Martonne Index | DMI (mm/°C) |
| Slope | Slope |
| Digital terrain model | DMT |

**Table S8.** Thermicity and Ombrothermic Indices according to Rivas-Martínez [27].

| Designation | Equation | Simbology |
|---|---|---|
| Thermicity Index (It; dimensionless) [21] | $It = (T + m + M)10$ | T – mean annual temperature (°C);<br>m – average minimum temperature of the coldest month (°C);<br>M – average maximum temperature of the coldest month (°C). |
| Ombrothermic Index (Io; dimensionless) | $Io = \left(\frac{Pp}{Tp}\right) \times 10$ | Pp – Yearly Positive Precipitation. In mm, the total average precipitation of those months whose average temperature is higher than 0°C;<br>Tp – Yearly Positive Temperature. In tenths of degrees Celsius, the sum of the monthly average temperature of those months whose average temperature is higher than 0°C. |

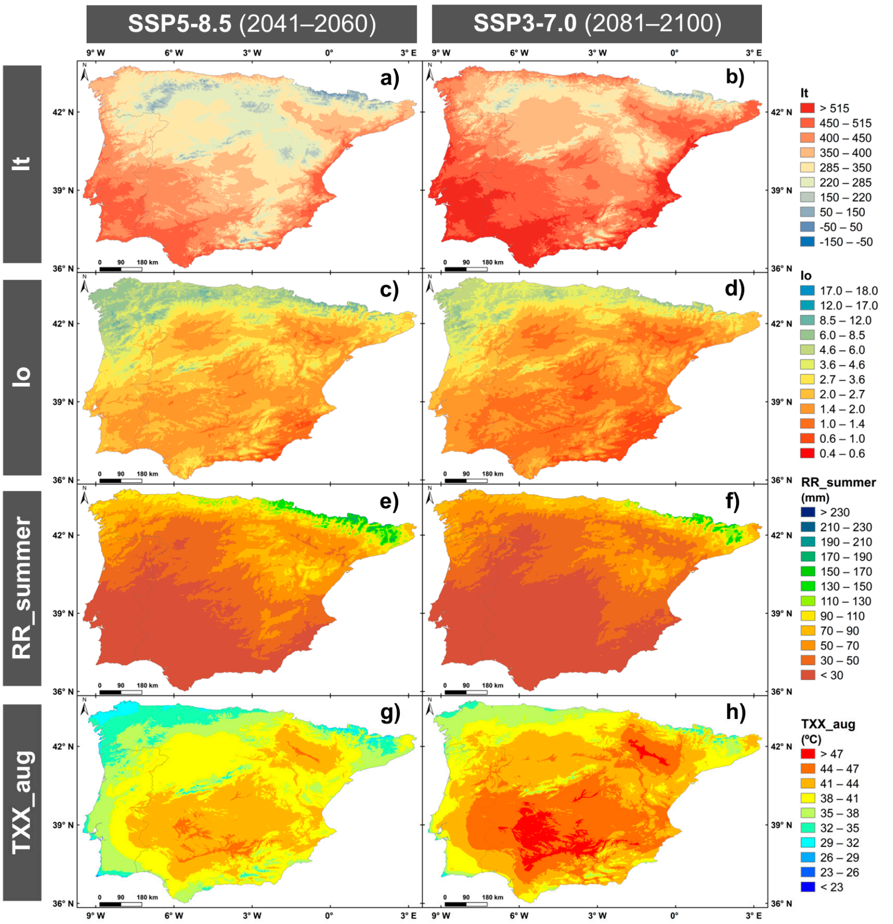


**Figure S1.** Ensemble means bio-ecological indicators for future scenarios: SSP5-8.5 (2041–2060) and SSP3-7.0 (2081–2100). The maps show: Thermicity index (It; **a**, **b**), Ombrothermic index (Io; **c**, **d**), Accumulated summer precipitation (June–August) in mm (RR_summer; **e**, **f**), and Maximum of the daily maximum temperature of the hottest month (August) in °C (TXX_aug; **g**, **h**). The threshold values for the thermotypes and ombrotypes horizons are defined by Rivas-Martínez [27].

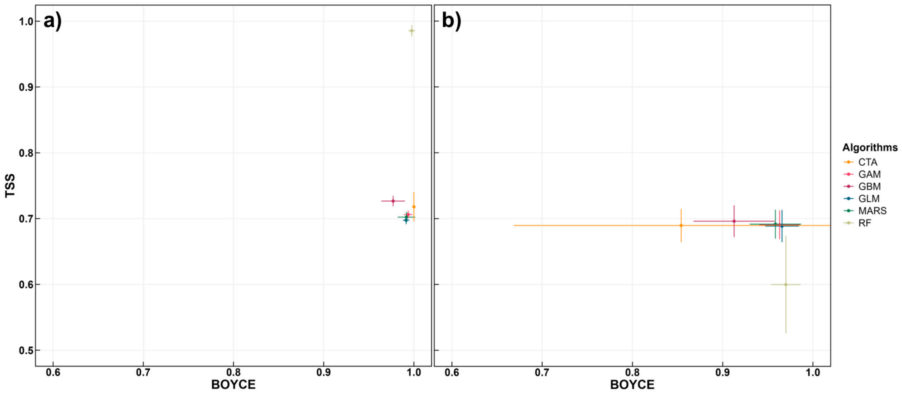


**Figure S2.** Calibration (**a**), validation (**b**) by algorithms: CTA, GAM, GBM, GLM, MARS and RF.

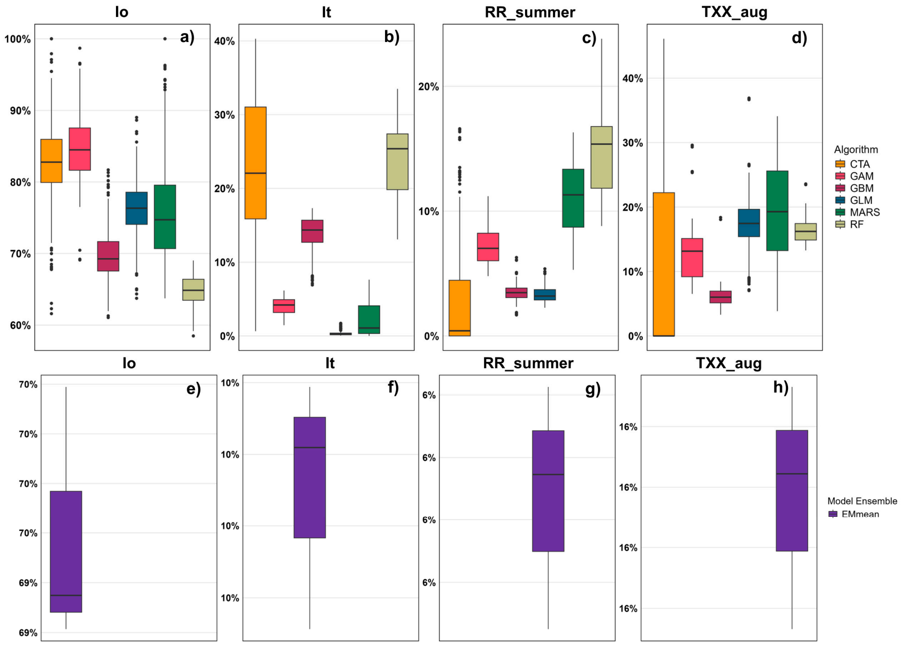

**Figure S3.** Bio-ecological indicators importance: Io (**a**, **e**), It (b, f), RR_summer (**c**, **g**) and TXX_aug (**d**, **h**), according to each algorithm and model ensemble, respectively.

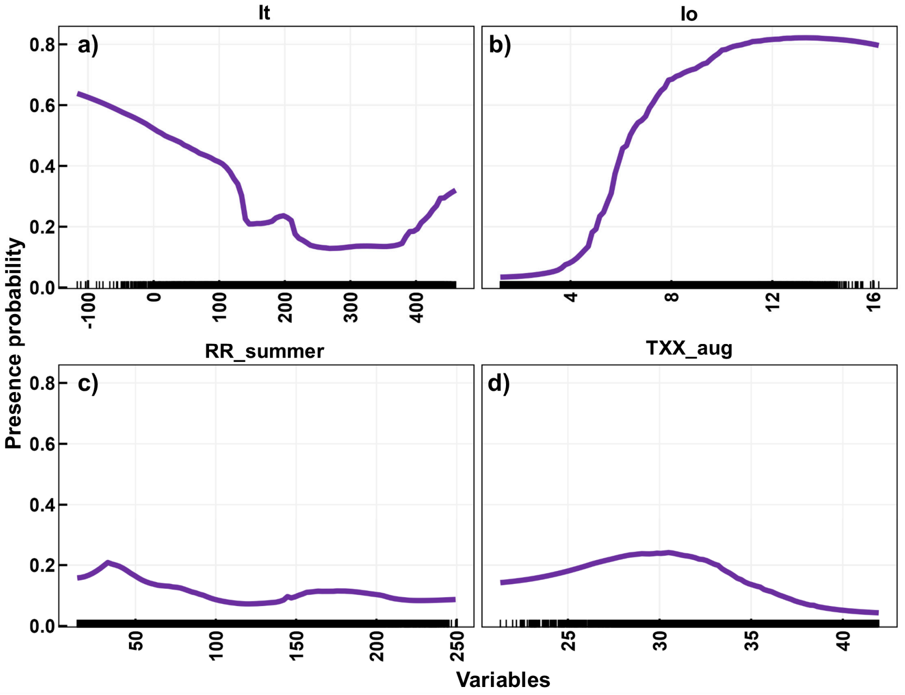

**Figure S4.** Response curves between bio-ecological indicators, It (**a**), Io (**b**), RR_summer (**c**) and TXX_aug (**d**), and the presence probability, in the model ensemble.

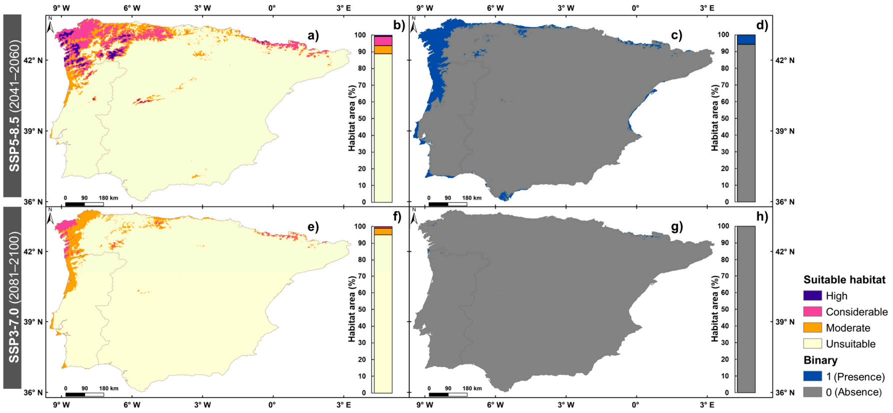


**Figure S5.** Climatic suitability classes (high, considerable, moderate and unsuitable) and binary distribution (1 = presence and 0 = absence) are represented for future scenarios: SSP5-8.5 for 2041–2060 (**a**, **c**) and SSP3-7.0 for 2081–2100 (**e**, **g**). The corresponding area percentages for a suitable habitat, based on suitability classes and binary classification, are presented for SSP5-8.5 (**b**, **d**) and SSP3-7.0 (**f**, **h**).

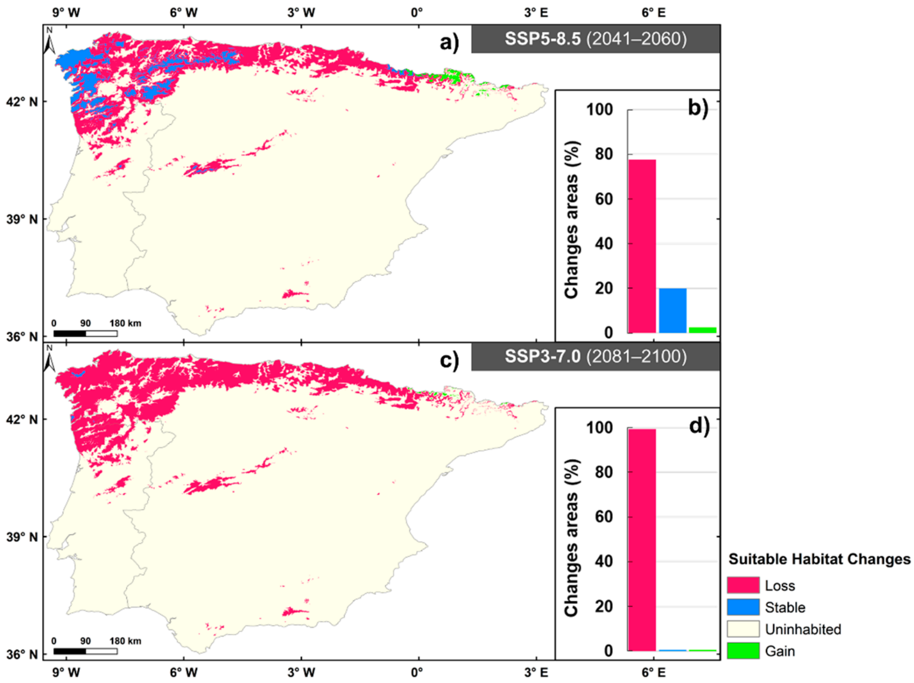


**Figure S6**. Dynamic changes in suitable areas between historical and future periods are illustrated. The changes are represented according to three categories: loss, stable and gain. These spatial distributions are represented in the maps, while their area percentage are presented in the graphs. Panels (**a**) and (**b**) correspond to SSP5-8.5 for 2041–2060, and panels (**c**) and (**d**) correspond to SSP3-7.0 for 2081–2100.

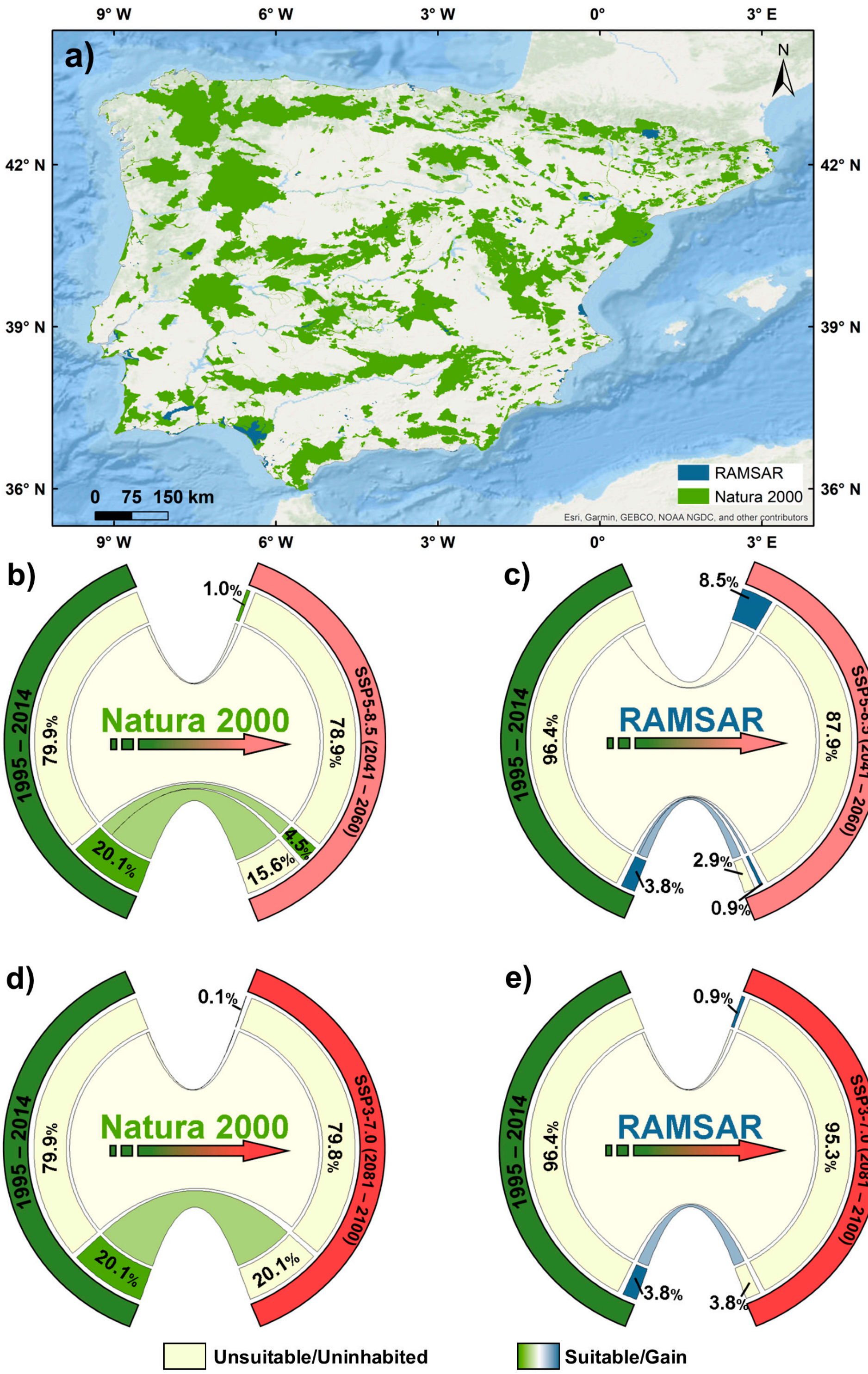


**Figure S7.** Natura 2000 and RAMSAR distribution in the Iberian Peninsula (**a**). Quantification of changes in suitability within Natura 2000 (**b**, **d**) and RAMSAR (**c**, **e**) areas, from the historical (1995–2014) period to future scenarios: SSP5-8.5 (2041–2060) and SSP3-7.0 (2081–2100).

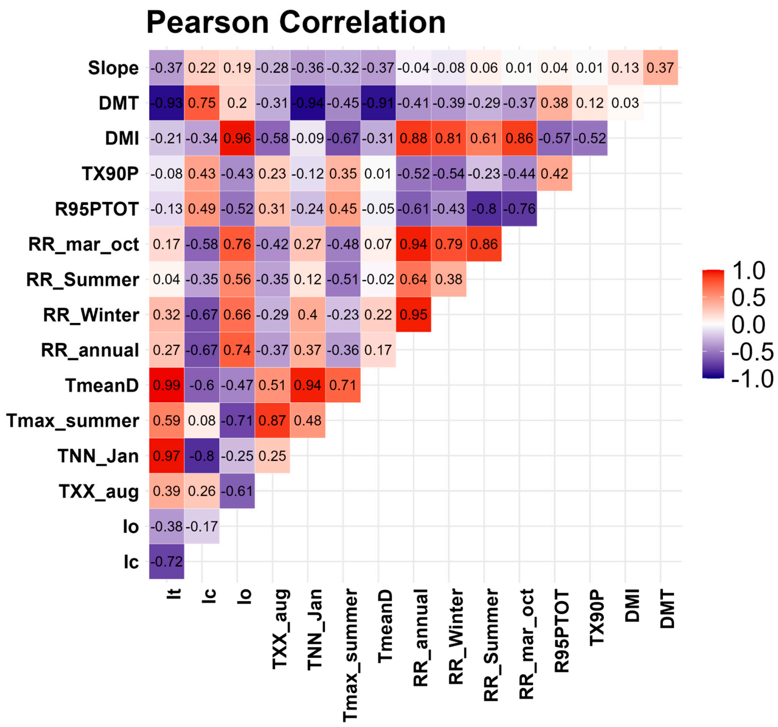


**Figure S8.** Pearson correlation for the 16 bio-ecological variables considered in the present study.
