# Supplementary figures and images for: Bio-Ecological Indicators for Gentiana pneumonanthe L. Climatic Suitability in the Iberian Peninsula
Source: Plants (Basel). 2025 Sep 12;14(18):2857. doi: 10.3390/plants14182857 (PMC12473960; doi:10.3390/plants14182857)

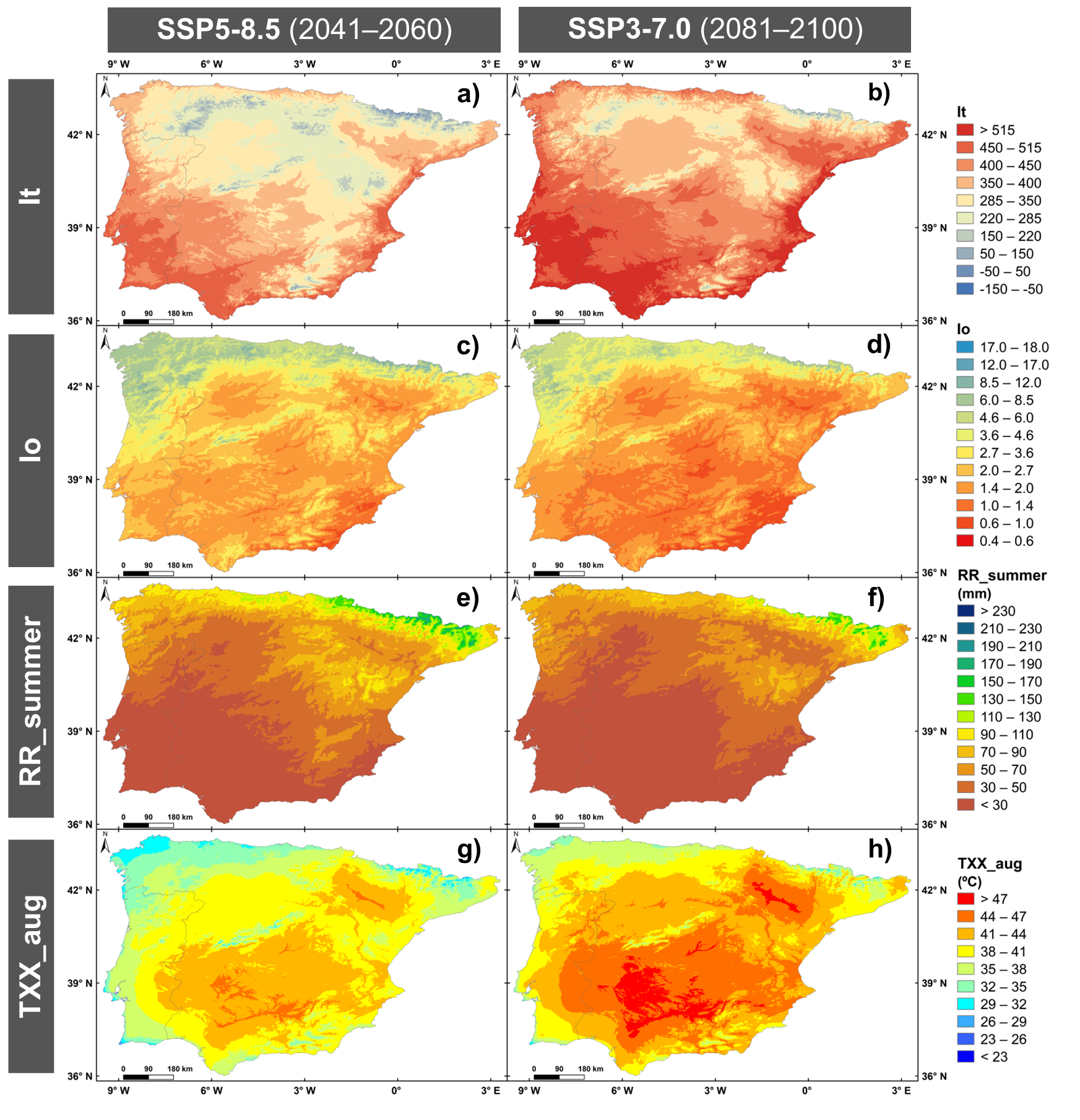

Supplement: Supplementary file 1 [file plants-14-02857-s001.zip › Figure SM1.png]

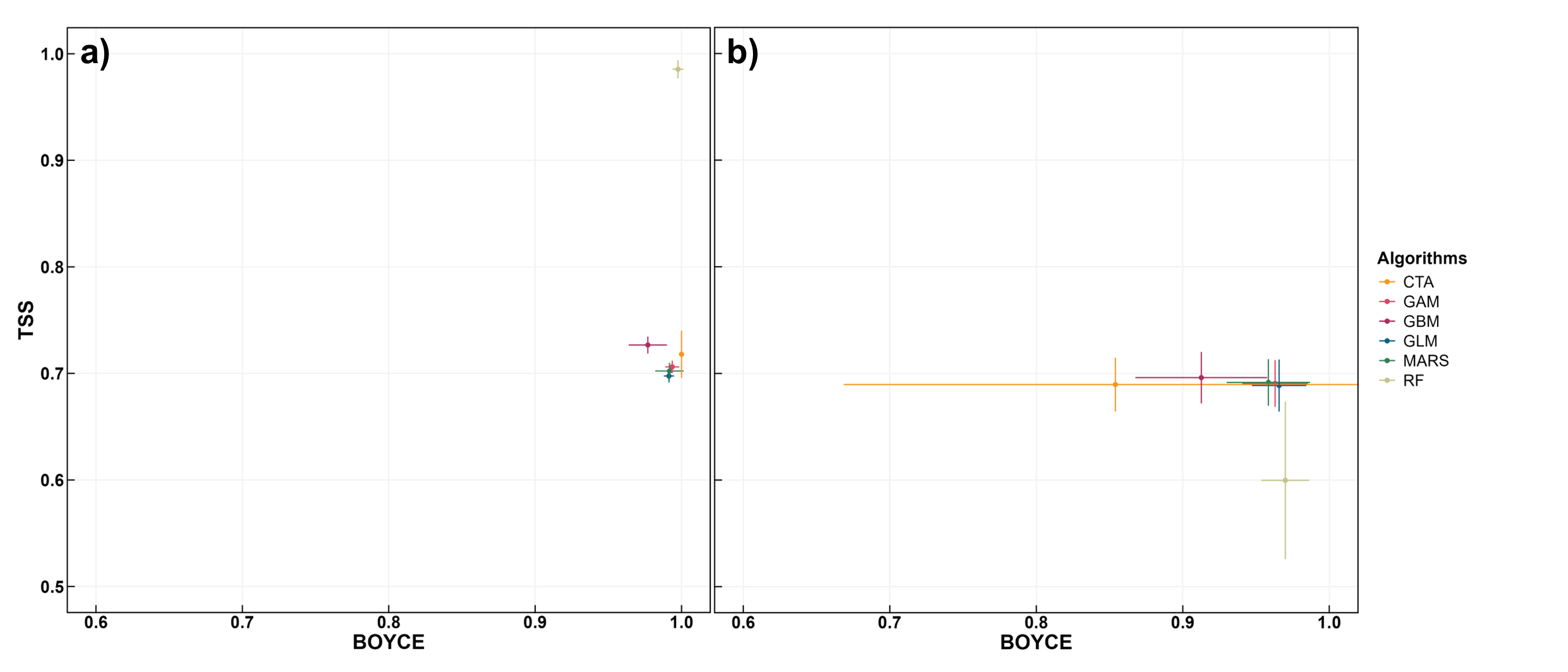

Supplement: Supplementary file 1 [file plants-14-02857-s001.zip › Figure SM2.png]

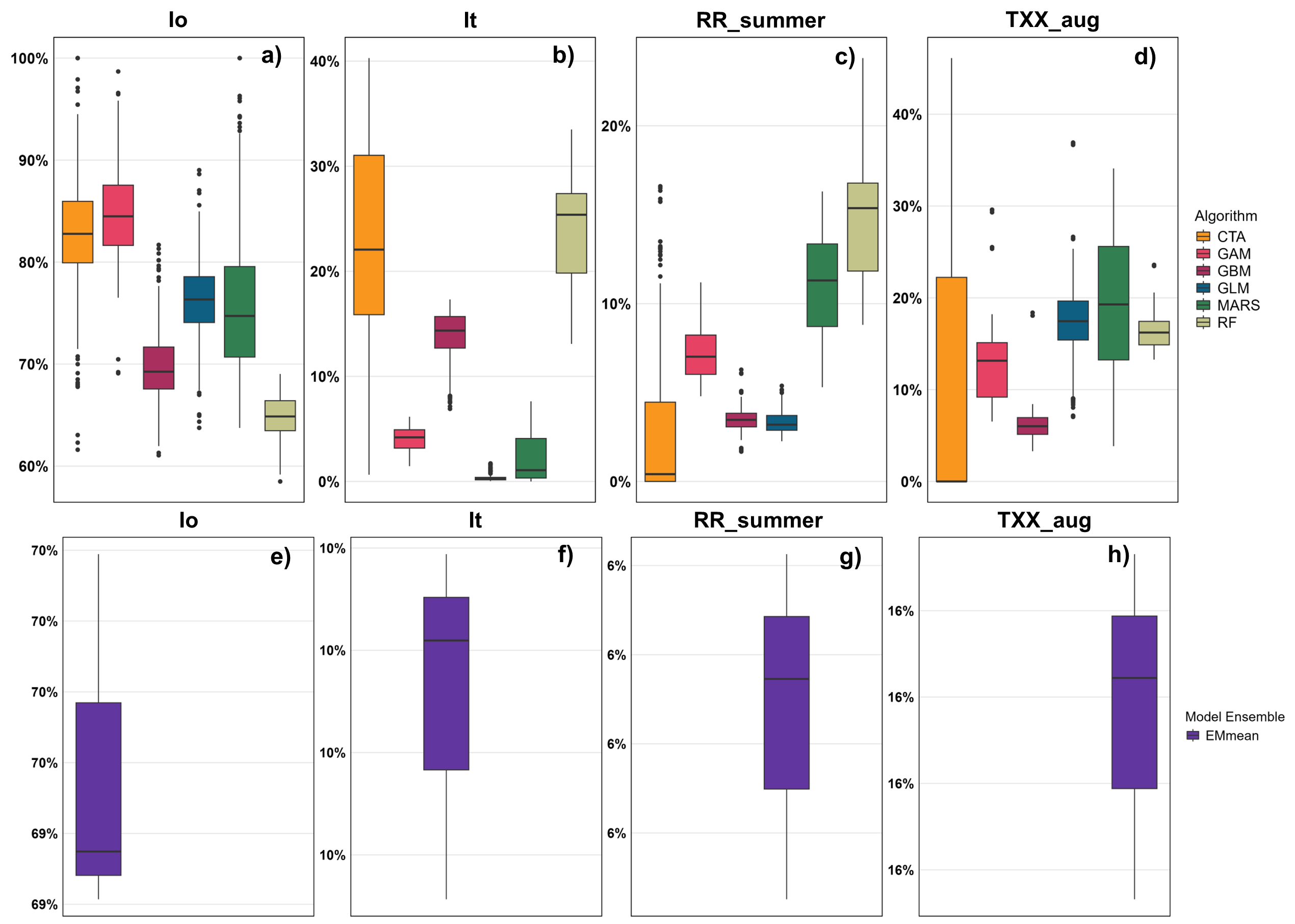

Supplement: Supplementary file 1 [file plants-14-02857-s001.zip › Figure SM3.png]

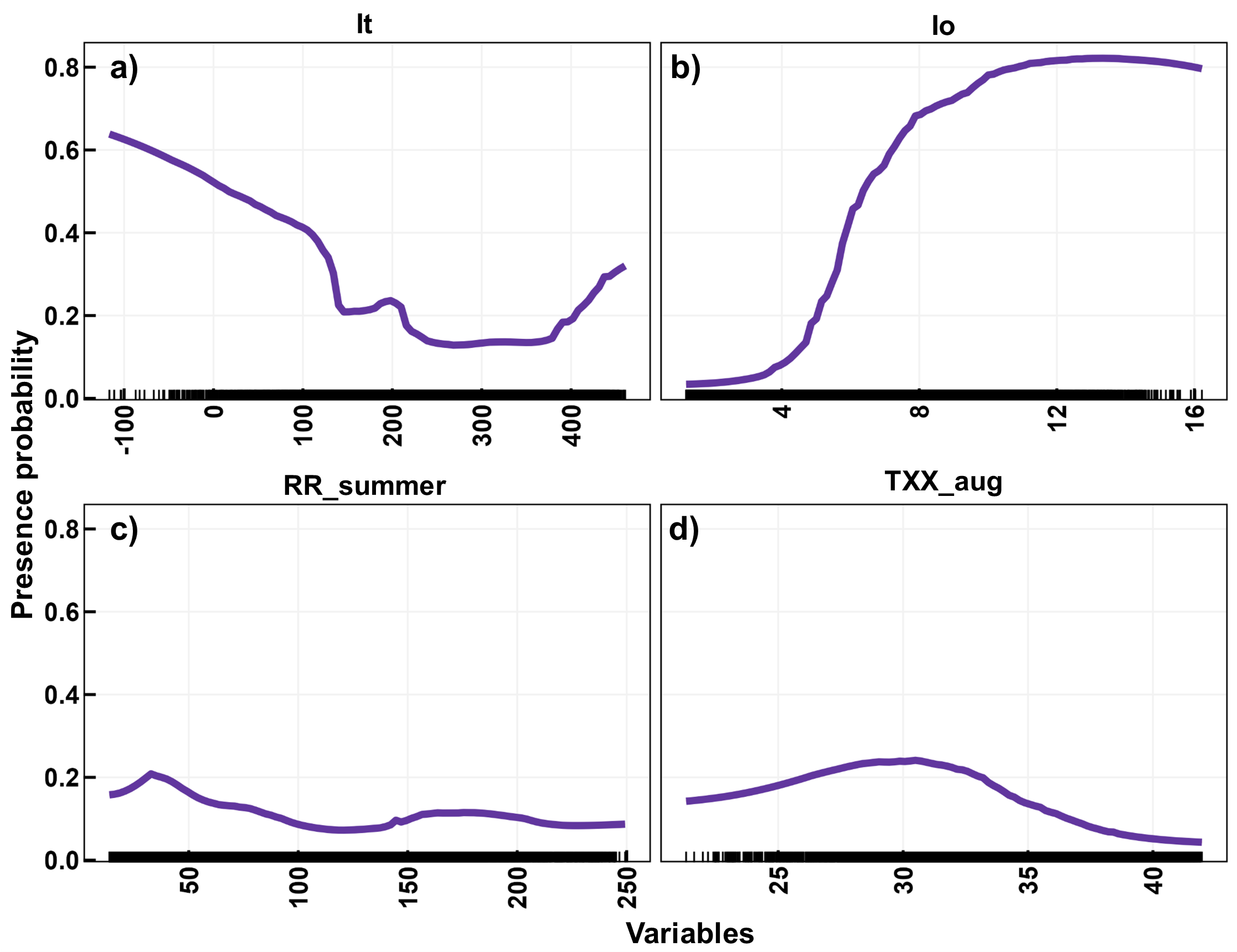

Supplement: Supplementary file 1 [file plants-14-02857-s001.zip › Figure SM4.png]

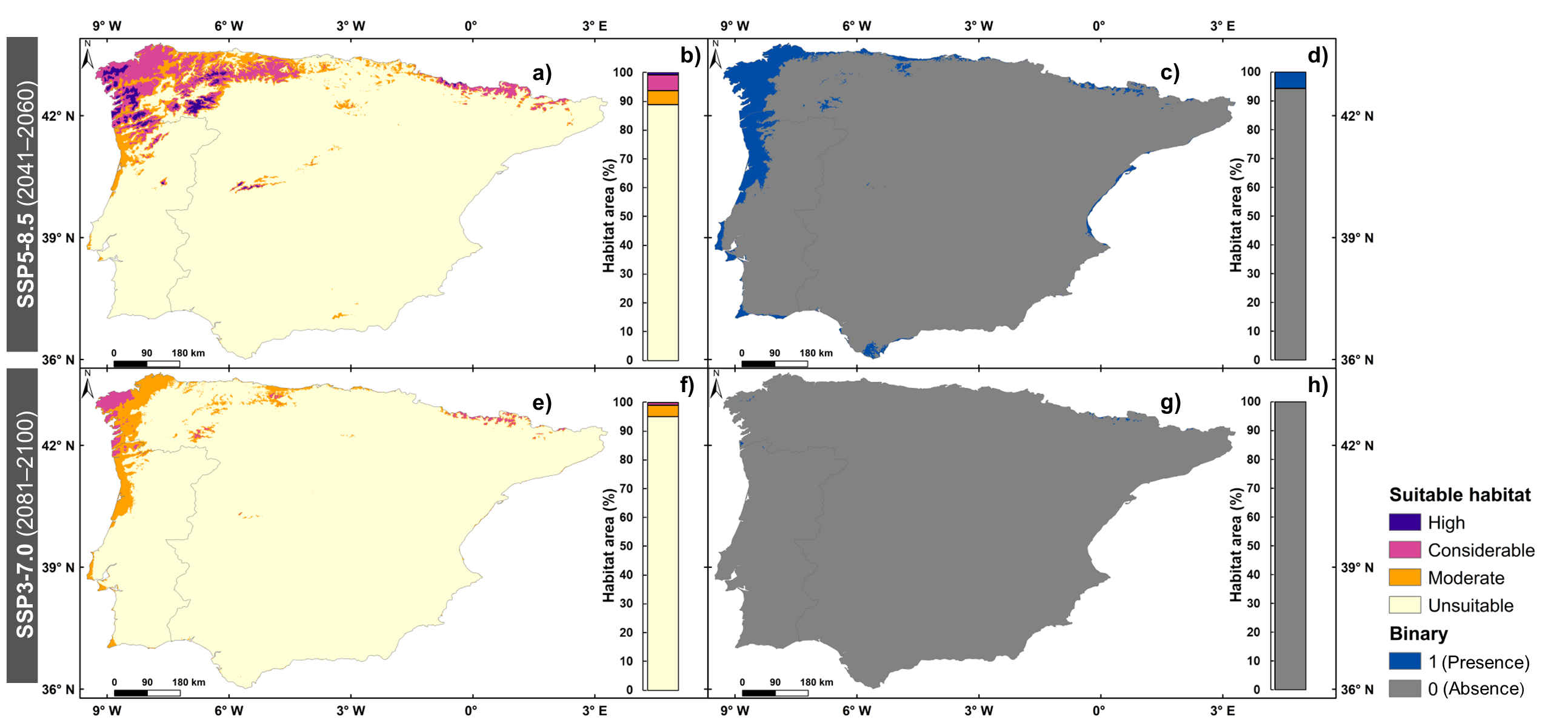

Supplement: Supplementary file 1 [file plants-14-02857-s001.zip › Figure SM5.png]

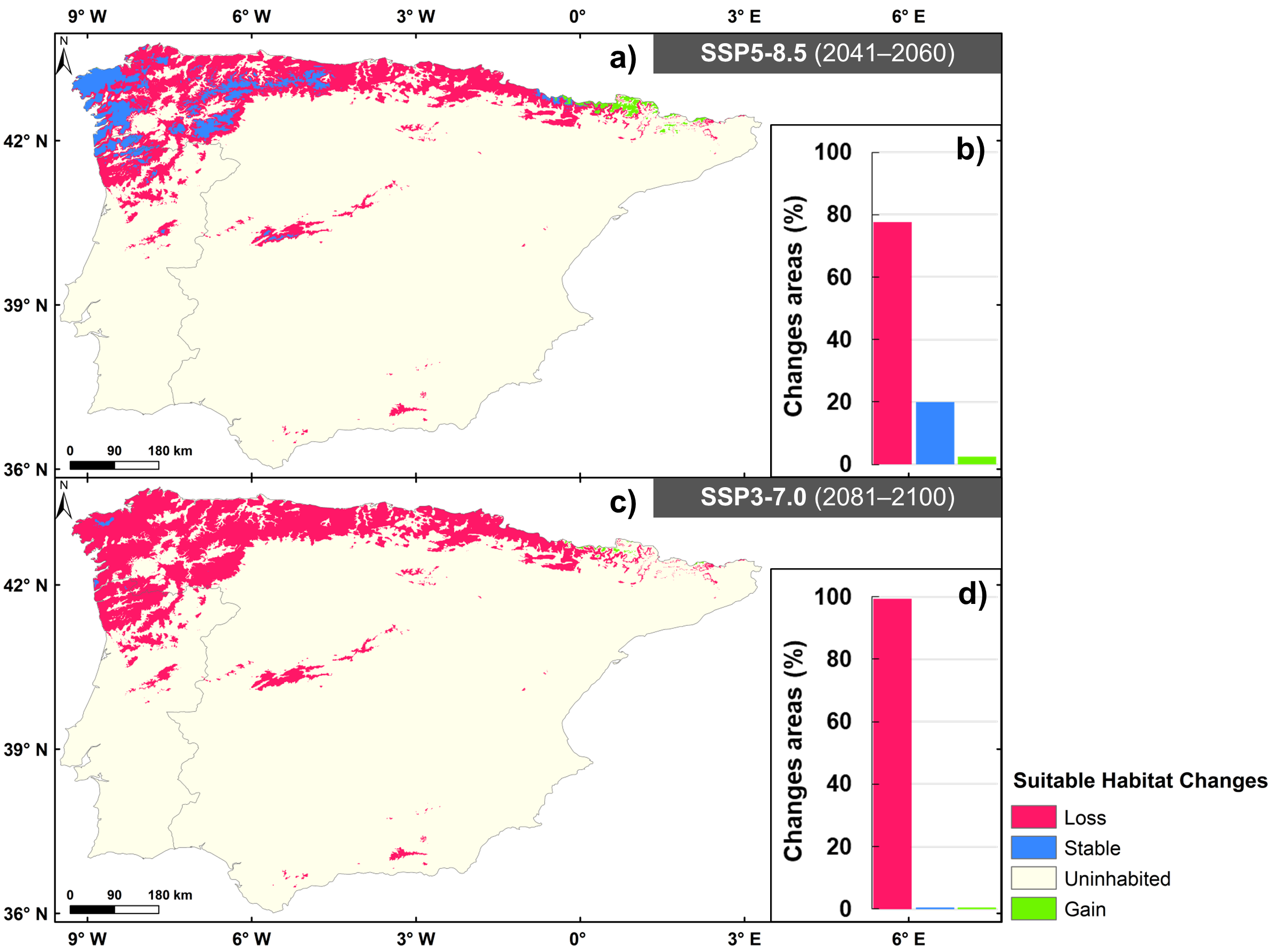

Supplement: Supplementary file 1 [file plants-14-02857-s001.zip › Figure SM6.png]

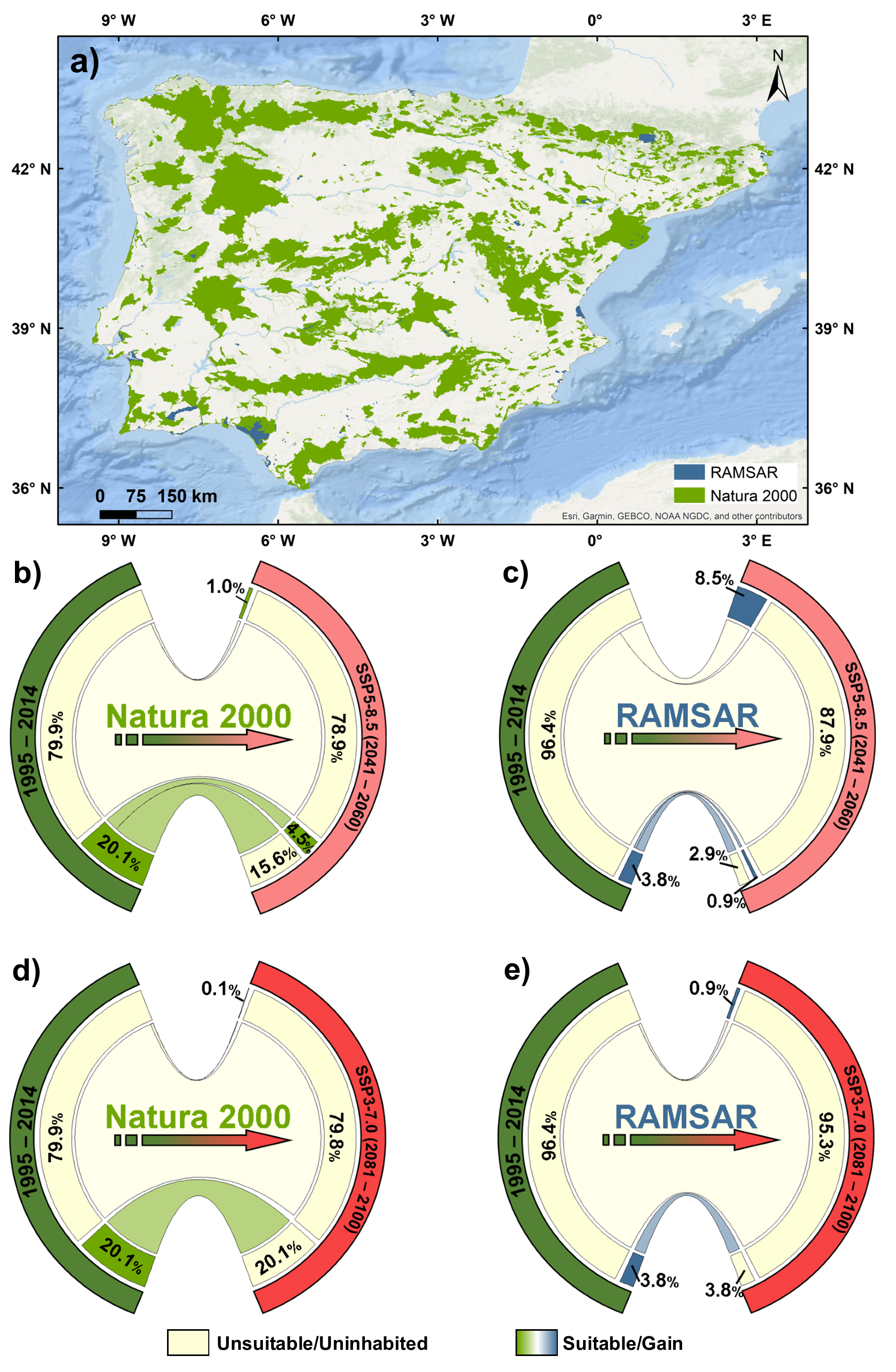

Supplement: Supplementary file 1 [file plants-14-02857-s001.zip › Figure SM7.png]

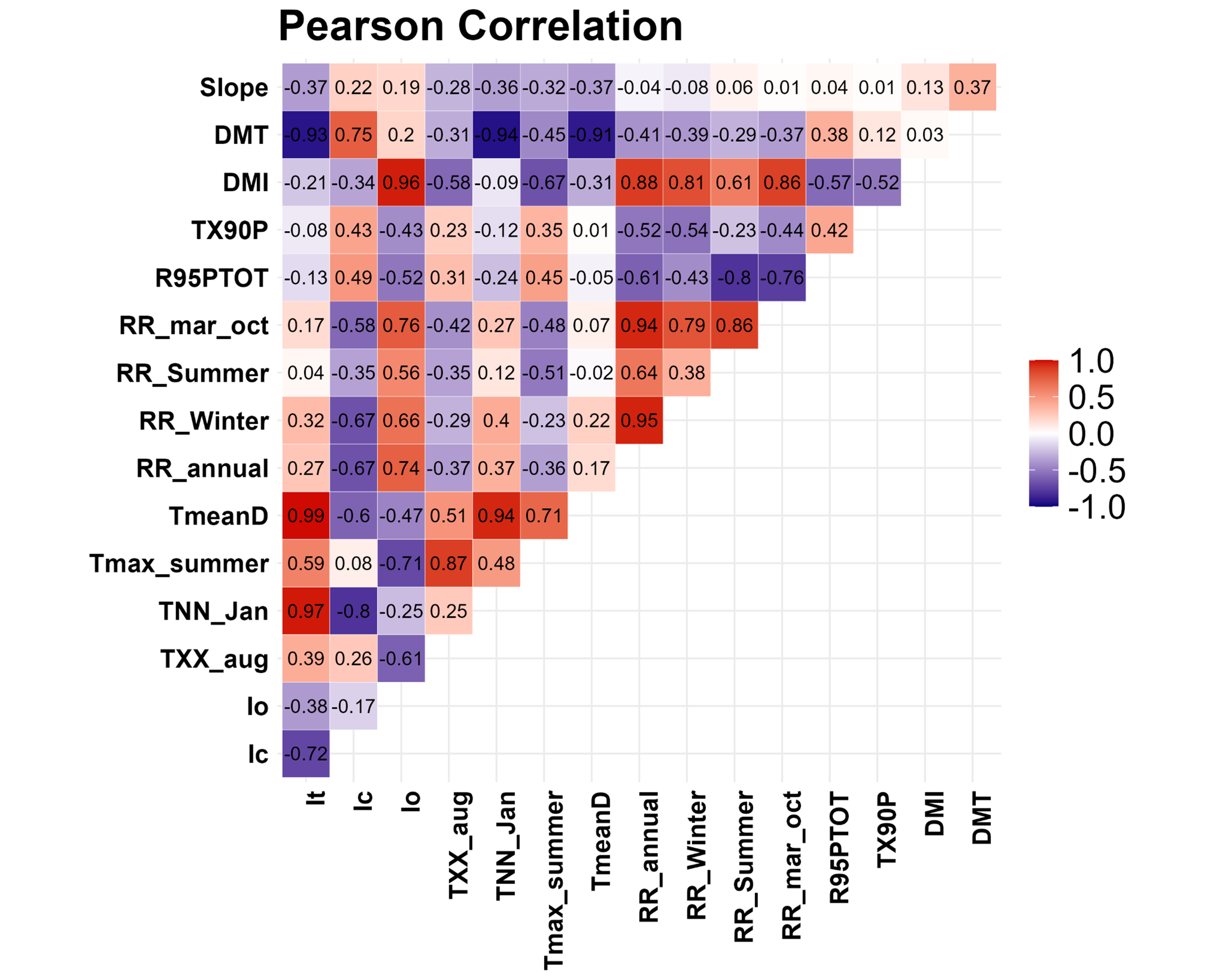

Supplement: Supplementary file 1 [file plants-14-02857-s001.zip › Figure SM8.png]
